# The dual role of autophagy in gynecologic malignancies: promoting and inhibiting

**DOI:** 10.3389/fcell.2026.1968563

**Published:** 2026-10-15

**Authors:** Dongying Gai, Ning Jia, Fanghua Qi, Lizhou Song, Wenwen Shao, Lin Zhao, Pingping Cai

**Affiliations:** 1 Department of Traditional Chinese Medicine, Shandong Provincial Hospital Affiliated to Shandong First Medical University, Jinan, China; 2 School of Anesthesiology, Naval Medical University, Shanghai, China

**Keywords:** autophagy, gynecologic malignancies, inflammatory microenvironment, tumor-promoting effects, tumor-suppressive effects

## Abstract

Autophagy is a fundamental lysosome-dependent degradation pathway that maintains cellular homeostasis by recycling intracellular components. It plays a critical role in stress adaptation, energy metabolism, and genomic stability. Emerging evidence supports that autophagy exhibits a context-dependent dual role in the development and progression of gynecologic malignancies. In the early stages of tumorigenesis or under specific molecular conditions, autophagy exerts tumor-suppressive effects by preserving genomic integrity, attenuating chronic inflammation, and promoting autophagy-related cell death. However, as tumors progress or are exposed to therapeutic stress—such as radiotherapy, chemotherapy, or targeted therapy—autophagy is frequently reactivated as an adaptive survival mechanism. This process enhances tumor cell tolerance to metabolic stress, oxidative damage, and therapeutic interventions, thereby facilitating tumor progression and contributing to drug resistance. Accordingly, this review systematically summarizes the molecular regulatory mechanisms of autophagy in gynecologic malignancies and its role in treatment responses. Furthermore, it highlights the therapeutic potential of targeting autophagy to improve precision treatment strategies.

## 1 Introduction

Gynecologic malignancies pose a significant threat to women’s health, with the most common types being cervical cancer (CC), endometrial cancer (EC), and ovarian cancer (OC), in addition to less frequent entities such as vulvar and vaginal cancers. Among these, CC, EC, and OC are the most prevalent and rank among the top ten cancers affecting women worldwide (Campo et al., 2015). In recent years, the incidence of gynecologic malignancies has steadily increased, driven by population aging, lifestyle changes, and persistent exposure to infectious risk factors. Early-stage CC and EC are associated with relatively high detection rates and favorable prognosis following standardized treatment (He and Li, 2021; Amant et al., 2005). In contrast, OC is characterized by insidious onset and the lack of specific early diagnostic biomarkers, with approximately 70% of patients diagnosed at advanced stages (stage III–IV), often accompanied by extensive peritoneal dissemination and distant metastasis (Stewart et al., 2019; Konstantinopoulos and Matulonis, 2023). Although vulvar and vaginal cancers are relatively rare, their development is closely associated with persistent human papillomavirus (HPV) infection and long-term chronic inflammation (Jhingran, 2022). Despite considerable advances in surgical techniques, chemoradiotherapy, targeted therapy, and immunotherapy, metastasis and recurrence remain formidable obstacles to improving patient outcomes. The 5-year survival rate for patients with advanced or drug-resistant disease remains below 20%, highlighting the limitations of current therapeutic strategies (Zhou et al., 2024). With the rapid evolution of molecular oncology, metabolomics, and tumor microenvironment (TME) research, it has become increasingly clear that gynecologic malignancies are not driven by single-gene alterations, but rather arise from complex disruptions in cellular homeostasis. As a central pathway regulating cellular homeostasis, autophagy has emerged as a key focus of research due to its involvement in tumor initiation, progression, metastasis, and therapeutic response.

Autophagy is a highly conserved lysosome-dependent intracellular degradation process that maintains cellular homeostasis and metabolic balance by removing damaged organelles, misfolded proteins, and intracellular pathogens (Glick et al., 2010). Based on substrate delivery mechanisms, autophagy can be classified into three major types: macroautophagy, microautophagy, and chaperone-mediated autophagy (CMA). Macroautophagy involves the formation of double-membrane autophagosomes that engulf cytoplasmic components and subsequently fuse with lysosomes for degradation and recycling (Li et al., 2021). In contrast, microautophagy directly engulfs cytoplasmic substrates through lysosomal membrane invagination or protrusion. CMA does not require vesicle formation; instead, it relies on chaperone proteins to recognize substrate proteins containing specific pentapeptide motifs, which are unfolded and translocated individually into the lysosomal lumen for degradation (Yamamoto and Matsui, 2024). Although these three pathways differ mechanistically, they ultimately converge on lysosome-mediated degradation and recycling. Under physiological conditions, autophagy plays a critical role in maintaining metabolic homeostasis, preserving mitochondrial function, enhancing antioxidant capacity, maintaining genomic stability, and preventing necrotic cell death. Consequently, it is involved in the pathogenesis and progression of multiple diseases, including cancer, neurodegenerative disorders, cardiomyopathies, diabetes, liver diseases, and autoimmune disorders (Glick et al., 2010).

Autophagy is not merely a cellular clearance mechanism but a highly dynamic regulatory system that plays a central role in tumor biology. Notably, autophagy exhibits stage-specific and context-dependent functions in gynecologic malignancies. During early tumorigenesis, autophagy functions as a protective mechanism by maintaining genomic integrity, facilitating cellular quality control, and mitigating oxidative stress, thereby suppressing tumor initiation and early progression (Camuzard et al., 2020; Barnard et al., 2016). However, during tumor progression and under therapeutic stress, autophagy provides metabolic advantages to tumor cells by sustaining energy production and promoting immune evasion, ultimately enhancing tumor survival and treatment resistance (Li et al., 2020; Gozuacik et al., 2017). Evidence from autophagy-deficient mouse models demonstrates that the accumulation of damaged organelles and persistent activation of inflammatory responses significantly promote tumorigenesis, highlighting the tumor-suppressive role of autophagy in inflammation-associated cancers (White et al., 2010). Conversely, studies in high-grade serous ovarian cancer (HGSOC) have shown that autophagy can be maintained independently of Beclin-1 (BECN1), suggesting that tumor cells can sustain autophagic activity through alternative pathways to adapt to stress and support tumor progression (Correa et al., 2015). Despite extensive research, most studies focus on individual tumor types or specific experimental systems, and a comprehensive understanding of autophagy across gynecologic malignancies remains lacking. Therefore, this review systematically delineates the molecular regulatory networks of autophagy and elucidates its dual role in tumor progression, aiming to provide a theoretical basis for future translational research and precision therapeutic strategies.

## 2 The fundamental mechanism of autophagy

### 2.1 Overview of key autophagy signaling pathways

In gynecologic malignancies, metabolic reprogramming of tumor cells and stress signals within the TME impose highly dynamic regulation on autophagy. Rather than functioning as isolated pathways, major autophagy-related signaling networks are interconnected and collectively integrate nutrient availability, cellular energy status, and stress signals to regulate autophagy initiation and adaptation. The mammalian target of rapamycin (mTOR) signaling pathway serves as a central inhibitory hub that suppresses autophagy initiation. The adenosine monophosphate (AMP)-activated protein kinase (AMPK) pathway mediates autophagy activation under energy-restricted conditions. The class III phosphatidylinositol 3-kinase (PI3K)–BECN1 complex is responsible for autophagy vesicle nucleation. The mitogen-activated protein kinase (MAPK)/p53 signaling axis participates in context-dependent regulation of autophagy under stress and therapy-related conditions. Through their coordinated interactions, these pathways enable autophagy to respond dynamically to changes in the cellular and tumor microenvironment. The following sections will elaborate on the molecular molecular mechanisms by which these signaling pathways govern autophagy.

#### 2.1.1 mTOR

The mTOR signaling pathway serves as a central hub integrating cellular metabolism and autophagy. In gynecologic malignancies, abnormal activation of the PI3K/protein kinase B (Akt) pathway, phosphatase and tensin homolog (PTEN) gene loss, and excessive growth factor signaling frequently lead to sustained hyperactivation of mechanistic target of rapamycin complex 1 (mTORC1). This hyperactivation inhibits autophagy initiation, thereby supporting rapid tumor cell proliferation and metabolic reprogramming. As the primary functional complex of mTOR, mTORC1 is activated under nutrient-rich conditions (Liu and Sabatini, 2020). Conversely, during tumor progression, conditions prevalent in the tumor microenvironment (TME)—such as hypoxia, nutrient deprivation, and energy stress—attenuate mTORC1 activity, relieving ULK1 inhibition and permitting autophagy induction (Kim et al., 2015). This dynamic regulatory mechanism enables tumor cells to maintain a homeostatic balance between nutrient supply and energy expenditure.

mTORC1 activation integrates multiple upstream signals. Its activity is jointly modulated by AMPK, PI3K/Akt, and Ras homolog enriched in brain (Rheb) signaling. AMPK promotes autophagy through two complementary mechanisms by directly phosphorylating ULK1 and inhibiting mTORC1, whereas the PI3K/Akt pathway suppresses autophagy by activating mTORC1 upstream (Dunlop and Tee, 2014). Amino acids can recruit mTORC1 to the lysosomal membrane by activating Ras-related GTP-binding protein guanosine triphosphatases, where mTORC1 interacts with Rheb and becomes activated, thereby inhibiting autophagy. Under amino acid deficiency, this process is disrupted, leading to rapid autophagy activation (Yu et al., 2010). These findings reveal the intricate regulatory mechanism of mTOR in sensing nutritional and energy signals during autophagy. Additionally, mTORC1 inhibits autophagy and suppresses the transcriptional expression of lysosomal biogenesis-related genes by phosphorylating the transcription factors transcription factor EB (TFEB) and transcription factor binding to IGHM enhancer 3 (TFE3), thereby preventing their nuclear translocation. Upon mTORC1 inhibition, TFEB/TFE3 are dephosphorylated and translocate to the nucleus, inducing transcription of autophagy and lysosomal-related genes, which enhances tumor cell adaptability to nutrient deprivation and metabolic stress (Bernard et al., 2020; Dang and Back, 2021; Yang et al., 2020). This pathway contributes to adaptive responses in various tumor cells under metabolic stress and therapeutic interventions and is closely linked to cancer cell survival and treatment tolerance.

Beyond mTORC1, mechanistic target of rapamycin complex 2 (mTORC2) also plays a crucial role in autophagy regulation. mTORC2 is composed of several core subunits, including mTOR, rapamycin-insensitive companion of mTOR (Rictor), mammalian stress-activated protein kinase-interacting protein 1 (mSIN1), and protein observed with Rictor (Protor). It primarily regulates cellular survival and metabolic homeostasis by phosphorylating downstream targets such as Akt, serum/glucocorticoid-regulated kinase 1 (SGK1), and protein kinase C (PKC) family members. Studies have shown that mTORC2 suppresses the transcription of autophagy-related genes mediated by forkhead box O (FOXO) transcription factors (FOXO1/3) through activation of Akt signaling, thereby indirectly reducing autophagy activity. Conversely, under conditions of energy stress or oxidative stress, decreased mTORC2 activity enhances FOXO-dependent autophagy responses, promoting cellular survival under adverse conditions (Kma and Baruah, 2022). Furthermore, in fibroblast models, prolonged autophagy induction accompanied by reactive oxygen species (ROS) accumulation has been shown to activate mTORC2, thereby promoting cellular senescence. This suggests that mTORC2 exerts bidirectional regulatory effects on autophagy and cellular fate decisions (Bernard et al., 2020).

Taken together, mTORC1 and AMPK play complementary roles in coordinating autophagy, with mTORC1 predominantly suppressing autophagy under nutrient-rich conditions, whereas AMPK promotes autophagy under energy-restricted conditions by inhibiting mTORC1 and activating ULK1.

#### 2.1.2 AMPK

AMPK serves as a pivotal molecule link between cellular energy status and autophagy regulation. When intracellular adenosine triphosphate (ATP) levels decrease and the AMP/ATP ratio increases, AMPK is rapidly activated. It promotes autophagy initiation by phosphorylating ULK1. Concurrently, AMPK suppresses fatty acid synthesis, protein synthesis, and multiple anabolic pathways downstream of mTORC1, thereby enabling cells to maintain essential metabolic demands under energy-restricted conditions (Herzig and Shaw, 2018). Furthermore, AMPK adapts to energy deprivation by modulating mitochondrial dynamics, oxidative phosphorylation, and substrate utilization, thereby helping tumor cells maintain metabolic flexibility and survival under hostile microenvironments (Maestri et al., 2024). In a HGSOC spheroid model, the calcium/calmodulin-dependent protein kinase kinase beta (CAMKKβ)-AMPK signaling pathway has been shown to be essential for maintaining autophagic flux and tumor cell survival, further underscoring the critical role of AMPK in stress adaptation and energy homeostasis in tumor cells (Laski et al., 2020).

In gynecologic malignancies, the TME is frequently characterized by sustained metabolic stress, including glucose deprivation, hypoxia, elevated lactate levels, and increased energy expenditure. These conditions drive persistent activation of AMPK-dependent autophagy, which serves as a crucial adaptive mechanism enabling tumor cells to redistribute energy, maintain energy homeostasis, and adapt to the adverse microenvironment (Yung et al., 2022; Tam et al., 2021). AMPK-regulated metabolic pathways are closely associated with tumor cell invasiveness, metastatic potential, and therapeutic responsiveness, suggesting that AMPK serves not only as a pivotal molecule in cellular metabolism and autophagy regulation but also as a potential therapeutic target in gynecologic malignancies (Tam et al., 2021).

#### 2.1.3 PI3K-BECN1

Beyond the mTOR–ULK1 axis, the class III PI3K-vacuolar protein sorting 34 (VPS34)- BECN1 protein complex plays an irreplaceable core role in autophagosome nucleation. BECN1, a key regulatory protein in autophagy, functions as a component of the class III PI3K (VPS34) complex. Its interaction with VPS34 is required for phosphatidylinositol 3-phosphate (PI3P) production and isolation membrane formation (Decuypere et al., 2012). BECN1 contains a Bcl-2 homology 3 (BH3) domain that interacts with anti-apoptotic proteins B-cell lymphoma 2 (Bcl-2)/B-cell lymphoma-extra large (Bcl-XL) via the BH3-groove to form an inhibitory complex. This complex restricts the nucleation function of the BECN1–VPS34 complex, representing a classic mechanism by which autophagy initiation is blocked (Levine et al., 2008). Under stress conditions, c-Jun N-terminal kinase (JNK)-1-mediated phosphorylation of Bcl-2 weakens its interaction with BECN1, thereby relieving BECN1 inhibition. Consequently, BECN1 reassembles with PI3K/VPS34 to form an active nucleation complex, initiating autophagosome formation (Wei et al., 2008).

In gynecologic malignancies, BECN1 expression correlates positively with autophagy markers such as microtubule and negatively with Bcl-2 expression levels, suggesting that decreased BECN1 expression may be accompanied by enhanced inhibitory complex formation and reduced autophagic activity. Moreover, high-grade tumors typically exhibit lower BECN1 expression levels (Valente et al., 2014). Meta-analysis results further indicate that BECN1 expression is closely associated with overall survival, progression-free survival (PFS), and early International Federation of Gynecology and Obstetrics (FIGO) stage in OC patients, suggesting its potential prognostic predictive value (Chen et al., 2020). Under stress or therapeutic conditions, BECN1 dissociates from inhibitory complexes and reassembles into the activated PI3K/VPS34 complex, serving as a crucial mechanism by which tumor cells initiate protective autophagy. This process influences autophagic flux, cell survival, and therapeutic response. However, it is noteworthy that in actual tumor cell models, BECN1 is not an absolute requirement for autophagy under certain conditions. Its specific role in autophagy regulation across different tumor contexts warrants further experimental investigation (Correa et al., 2015).

#### 2.1.4 MAPK/p53

Members of the MAPK family including extracellular signal-regulated kinase (ERK), JNK, and p38 mitogen-activated protein kinase play crucial roles in regulating autophagy in response to oxidative stress, inflammatory stimuli, and deoxyribonucleic acid (DNA) damage. As a core mediator, JNK interferes with the binding of Bcl-2 to BECN1 through multi-site phosphorylation of Bcl-2, thereby releasing BECN1 inhibition and subsequently promoting autophagosome nucleation and autophagy initiation (Park et al., 2009). p53 exhibits bidirectional, context: nuclear p53 induces autophagy by transcribing genes such as damage-regulated autophagy modulator 1 (DRAM1), while cytoplasmic p53 suppresses the process (Lorin et al., 2010). In gynecologic malignancies, platinum-based chemotherapy, radiotherapy, and other DNA-damaging therapies frequently activate the MAPK/p53 autophagy axis. This pathway influences tumor cell sensitivity to treatment and the evolution of drug resistance by regulating autophagic flux (Ma et al., 2025), providing a molecular basis for understanding the adaptive responses of gynecologic malignancies to therapeutic stress.

Overall, autophagy regulation is not dominated by a single signaling pathway but is accomplished through the coordinated action of multiple signaling networks. In gynecologic malignancies, mTORC1 and mTORC2 form the core hubs regulating autophagy, while the AMPK energy-sensing pathway, the PI3K–BECN1 complex, and the MAPK/p53 signaling axis contribute to the initiation and fine-tuning of autophagy under different stress conditions. The mechanisms underlying these four common autophagy pathways are illustrated in Figure 1.

**FIGURE 1:**
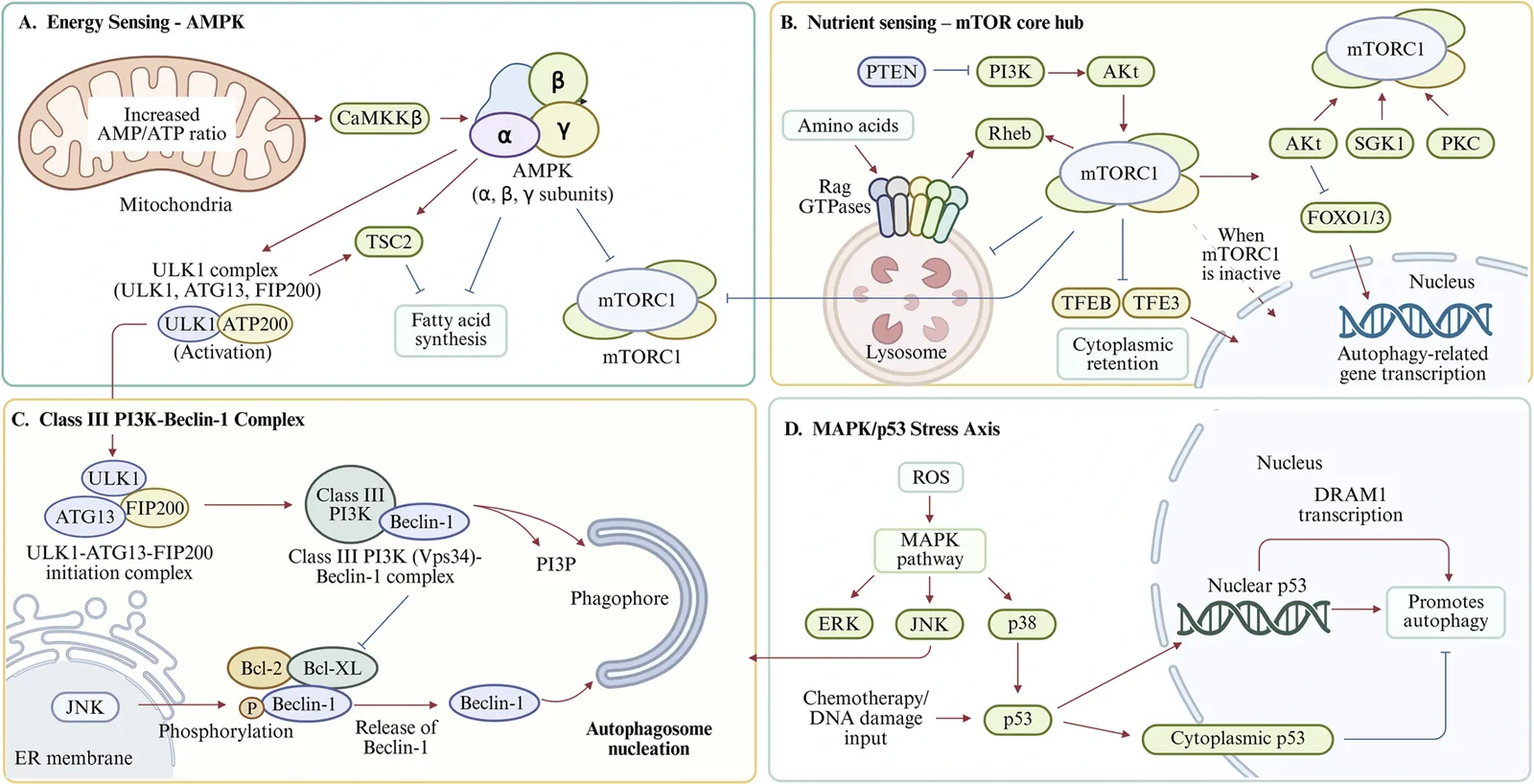
Four signaling pathways regulating autophagy in gynecologic malignancies. **(A)** When energy is insufficient (AMP/ATP ratio increases), CAMKKβ activates AMPK. AMPK directly phosphorylates ULK1 to initiate autophagy and inhibits mTORC1 and anabolic processes, thereby aiding cellular survival under energy-restricted conditions. **(B)** When nutrients and growth factors are abundant, PI3K/Akt and amino acid signaling activate mTORC1, inhibiting ULK1 and TFEB/TFE3 to block autophagy. Under hypoxic or nutrient-deprived conditions, mTORC1 activity decreases, leading to activation of ULK1 and TFEB/TFE3, thereby enhancing autophagy. mTORC2 indirectly regulates autophagy-related gene expression through the Akt–FOXO axis. **(C)** BECN1 is inhibited by Bcl-2/Bcl-XL and cannot form a nucleation complex with VPS34; under stress, JNK phosphorylates Bcl-2, releasing BECN1 to promote PI3P generation and autophagosome nucleation. **(D)** ROS, chemotherapy, and DNA damage activate the MAPK pathway. JNK promotes BECN1 release to enhance autophagy; p53 exerts a dual effect, with nuclear p53 promoting autophagy while cytoplasmic p53 inhibits it.

### 2.2 The relationship between autophagy and cellular metabolism

Cellular metabolism is the core process sustaining life activities and internal homeostasis, encompassing energy production, material synthesis, and biomolecule recycling. Under physiological conditions, glucose serves as the primary cellular energy source, continuously generating ATP through glycolysis, the tricarboxylic acid cycle (TCA), and mitochondrial oxidative phosphorylation. This energy fuels essential processes such as biosynthesis, transmembrane transport, and signal transduction (Bonora et al., 2012; Liemburg-Apers et al., 2011). In gynecologic malignancies, rapid tumor cell proliferation leads to a dramatic increase in energy demand, hypoxia and nutrient deprivation within the TME, and metabolic disruption caused by therapeutic interventions. These factors heighten the dependence of tumor cells on metabolic regulation and substrate recycling, positioning autophagy as an increasingly vital adaptive pathway for maintaining energy homeostasis and conferring a survival advantage.

When intracellular ATP production is restricted, the AMP/ATP ratio increases, or energy demand surges, autophagy is rapidly activated. It degrades amino acids, fatty acids, and damaged organelles to provide alternative substrates for metabolic pathways, and, under certain conditions, assists in maintaining or restoring ATP production (Bressan et al., 2020). This process is particularly crucial in tumor cells, as it alleviates energy crises while providing the material basis for biosynthesis and proliferation. For instance, in an *in vitro* osteoblast model, mechanical loading-induced energy expenditure leads to a significant drop in ATP levels and reduced cell viability when autophagy is inhibited, underscoring the role of autophagy in maintaining ATP homeostasis under high energy demand. In migrating neural progenitor cells *in vivo*, dynamic fluctuations in the ATP/adenosine diphosphate (ADP) ratio directly signal autophagy induction, which in turn sustains energy supply required for cell migration. Recent studies have revealed that the dual-FYVE domain protein double FYVE-containing protein one possesses ATPase activity, with its ATP hydrolysis capacity serving as a key regulator of omegasome formation and selective autophagy occurrence. These findings collectively demonstrate that autophagy is not merely an energy-consuming process but an active adaptive mechanism tightly coupled with ATP sensing and energy regulation (Bressan et al., 2020; Zhang et al., 2018; N Hse et al., 2023).

In terms of energy metabolism, autophagy responds rapidly to nutrient deprivation by degrading proteins, lipid droplets, and glycogen, releasing amino acids, fatty acids, and glucose that feed into the TCA cycle and oxidative phosphorylation to sustain ATP production (MacLean et al., 2023). Among these, mitophagy plays a crucial role in gynecologic malignancies. It maintains mitochondrial quality and oxidative metabolic efficiency by clearing damaged mitochondria, helping tumor cells adapt to hypoxic environments and reducing the accumulation of excessive reactive oxygen species (ROS).

When intracellular ATP levels decrease and the AMP/ATP ratio increases, AMPK is activated. AMPK promotes autophagy initiation by phosphorylating ULK1 while simultaneously inhibiting energy-consuming processes such as fatty acid synthesis and protein synthesis, as well as mTORC1-mediated anabolic pathways. This dual action enables cells to maintain essential metabolic demands and homeostasis under energy-restricted conditions (Oduro et al., 2022). Additionally, AMPK facilitates the selective clearance of damaged mitochondria via autophagy-related mechanisms, sustaining oxidative phosphorylation efficiency and energy output. Simultaneously, it regulates substrate utilization patterns to adapt to energy deprivation, thereby mitigating metabolic stress-related damage in tumor cells under hypoxic or nutrient-deprived conditions (Singh et al., 2009). Analysis of clinical samples has revealed significantly reduced expression of the AMPK β1 subunit during OC progression., a phenotype asscociated with increased tumor invasiveness and poor clinical outcomes. This further supports the AMPK pathway as a core driver of tumor metabolic reprogramming in the progression of gynecologic malignancies (Li et al., 2014).

Beyond energy supply, autophagy also plays a crucial role in maintaining material metabolism balance within tumor cells. Amino acid autophagy provides nitrogen sources for protein resynthesis under starvation; lipophagy releases fatty acids by degrading lipid droplets, supplying substrates for mitochondrial β-oxidation. This mechanism is vital for sustaining lipid homeostasis and energy supply in tumor cells (Feng et al., 2024). Under glucose deprivation or other stress conditions, autophagy also promotes glycogen breakdown and maintains the availability of substrates for glycolysis. Some studies suggest that autophagy may further regulate carbohydrate metabolism by modulating specific enzymes or membrane structures (Xu and Fan, 2022).

In summary, autophagy serves as a central hub in metabolic regulation of gynecologic malignant tumor cells through degradation and recycling mechanisms. It acts not only as a crucial compensatory pathway during energy deprivation but also orchestrates amino acid, lipid, and glucose metabolism to maintain material homeostasis. This enables tumor cells to sustain essential life activities and proliferative capacity even under metabolic stress. Overall, autophagy coordinates the recycling of multiple metabolic substrates with the maintenance of mitochondrial homeostasis, thereby supporting tumor-cell metabolic adaptation and survival under nutrient or energy stress.

### 2.3 The role of autophagy in cellular stress responses

During tumorigenesis and progression, cells are continuously subjected to multiple exogenous and endogenous stressors, including oxidative stress, endoplasmic reticulum (ER) stress, DNA damage, hypoxia, nutrient deprivation, and drug exposure. In gynecologic malignancies, these stressors originate not only from rapid tumor cell proliferation and metabolic dysregulationbut are also from the TME and therapeutic interventions. In response, autophagy plays a pivotal role in maintaining cellular homeostasis and promoting adaptive survival of tumor cells by clearing damaged organelles, toxic protein aggregates, and invading pathogens (Kitada and Koya, 2021). Different stressors regulate autophagy through specific signaling pathways, thereby contributing to protective adaptation, metabolic remodeling, and cell fate determination.

Oxidative stress is one of the most common inducers of autophagy factors in gynecologic malignancies. During the development of ovarian, cervical, and EC, tumor cells are frequently exposed to high levels of ROS triggered by treatment-related stimuli such as prolonged hypoxia, metabolic abnormalities, platinum-based chemotherapy, and radiation therapy. Excessive ROS cause oxidative damage to mitochondria, lipids, and DNA. Tumor cells can eliminate damaged mitochondria through autophagy, particularly mitophagy, thereby blocking the sustained ROS accumulation and avoiding apoptosis or necrosis-level cascading damage (Wang B. et al., 2023). Simultaneously, ROS act as signaling molecules that promote autophagosome formation by activating AMPK or inhibiting mTOR, thereby releasing suppression of the autophagy initiation complex and helping tumor cells maintain metabolic homeostasis under sustained oxidative stress (Fang et al., 2021; Li et al., 2015). At the molecular level, ROS enhances the activity of the ULK1 and VPS34–BECN1 complex, promoting the initiation and expansion of isolation membranes. Morver, oxidative modification of key autophagy proteins such as ATG4 and LC3 improves the membrane-binding efficiency of the lipidated LC3 (LC3-II), accelerating autophagosome maturation (Zhou et al., 2022; Li et al., 2019). Additionally, ROS can induce the expression of multiple autophagy-and antioxidant-related genes through the kelch-like ECH-associated protein 1 (Keap1)–nuclear factor erythroid 2-related factor 2 (Nrf2) signaling axis, serving as a crucial mechanism for tumor cells to resist oxidative damage, adapt to therapeutic stress, and maintain survival (Chen et al., 2024). The above evidence suggests that ROS-mediated autophagy regulation plays a significant role in the development, progression and treatment response of gynecologic malignancies.

ER stress is also highly active in gynecologic malignancies. Rapid proliferation of tumor cells and elevated protein synthesis significantly increase ER load, leading to sustained activation of the unfolded protein response (UPR). The UPR regulates misfolded protein processing through three classical pathways—protein kinase R-like ER kinase (PERK), activating transcription factor 6 (ATF6), and inositol-requiring enzyme 1 (IRE1)—and activates autophagy under sustained stress to alleviate ER pressure (Qi and Chen, 2019). Within the PERK pathway, the PERK–eukaryotic initiation factor 2 alpha (eIF2α)–activating transcription factor 4 (ATF4) axis upregulates autophagy-related genes such as LC3 and ATG12, enhancing autophagy capacity to maintain protein quality control (B’Chir et al., 2013; Yang et al., 2013). This mechanism further promotes the clearance of misfolded proteins and damaged organelles, serving as a crucial adaptive strategy for gynecologic malignant tumor cells under sustained ER stress. The IRE1–JNK pathway, by weakening Bcl-2-mediated inhibition of BECN1, facilitates the formation of the autophagy initiation complex, thereby enhancing autophagic flux under stress conditions (Rashid et al., 2015; Ren et al., 2021). This has been validated in multiple gynecologic malignancy models, particularly under treatment-induced ER stress, where it help tumor cells buffer protein folding stress and delay cell death (Flindris et al., 2025).

Hypoxia is a hallmark feature of the gynecologic malignant TME, particularly pronounced in OC peritoneal metastases and deep regions of solid tumors. Under hypoxic conditions, hypoxia-inducible factor 1-alpha (HIF-1α) upregulates mitochondrial autophagy receptors such as Bcl-2/adenovirus E1B 19 kDa interacting protein 3 (BNIP3) and BCL2 interacting protein three like (NIX) (Madhu et al., 2020; Bellot et al., 2009), accelerate the clearance of damaged mitochondria and reduce excess ROS generated by oxidative phosphorylation, helping tumor cells adapt to hypoxic environments and maintain energy homeostasis (Wang et al., 2017).

DNA damage stress holds particular pathological and therapeutic significance in gynecologic malignancies. On one hand, the highly proliferative state of tumor cells readily induces DNA replication stress and structural damage. On the other hand, platinum-based chemotherapeutic agents such as cisplatin exert their antitumor effects by inducing DNA intrastrand and interstrand crosslinksand are widely used as first-line treatments for OC and certain EC (Takatori et al., 2012; Wynne et al., 2007). Under DNA damage conditions, p53 and its downstream signaling pathways activate autophagy, providing a time window for DNA repair by clearing damaged proteins or promoting cell cycle arrest (Ryan, 2011). A positive feedback loop exists between p53 and autophagy:p53-induced autophagy enhances cellular survival under genotoxic stress and improves DNA repair efficiency (White, 2016). Furthermore, in DNA damage-induced cellular senescence, the transcription factor GATA binding protein 4 (GATA4)—a selective autophagy substrate—accumulates stably due to impaired degradation mediated by sequestosome 1/p62, thereby further regulating post-stress cellular fate (Kang et al., 2015).

It is noteworthy that autophagy plays a dual role in tumor cell stress responses: under mild or transient stress conditions, autophagy exerts a protective effect by maintaining homeostasis, clearing damage, and regulating metabolism; whereas under extreme or persistent stress, autophagy may become overactivated and contribute to cell death (D’Arcy, 2019). Furthermore, significant interactions exist between different stress pathways. For instance, oxidative stress frequently accompanies ER stress, while hypoxia can simultaneously induce energy stress and increased ROS production. Autophagy serves as a coordinating hub in these cross-signaling events, enabling tumor cells to achieve adaptive remodeling within fluctuating microenvironments. Overall, within the context of gynecologic malignancies, autophagy functions as a central regulatory mechanism of cellular stress responses. It integrates multiple signaling pathways to counteract endogenous and exogenous stresses, ensuring tumor cell survival and homeostasis through diverse functions including metabolic redistribution, damage repair, and fate regulation within dynamic stress environments. The adaptive integration functions of autophagy under various stress conditions are systematically presented in Figure 2.

**FIGURE 2:**
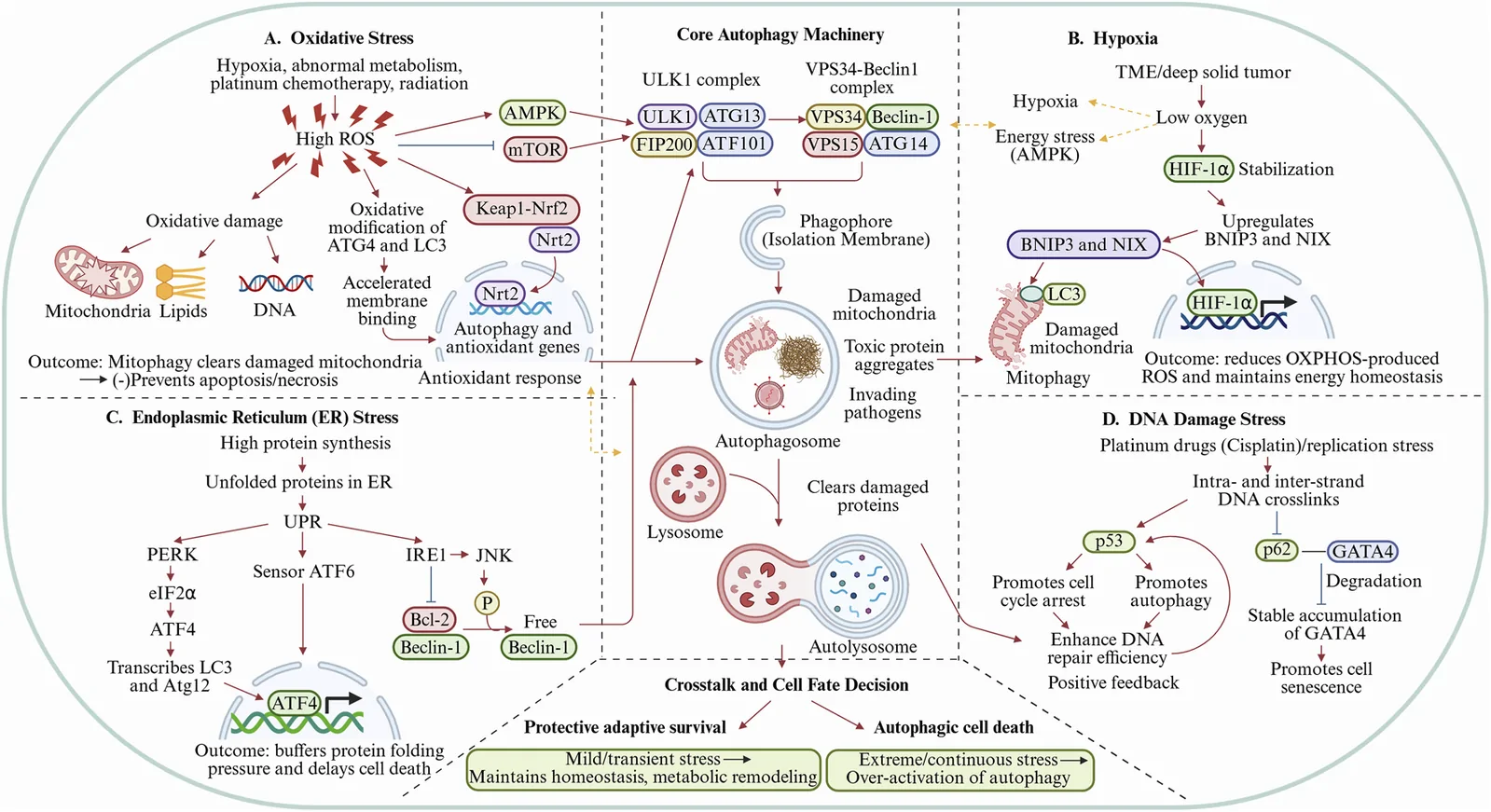
Multidimensional Regulatory Role of Autophagy in Stress Responses of Gynecologic Malignant Tumor Cells. **(A)** Oxidative Stress: Stimuli such as hypoxia and abnormal metabolism induce oxidative damage to mitochondria, lipids, and DNA, leading to a significant increase in intracellular ROS levels. This activates AMPK and inhibits mTOR, mediating mitochondrial autophagy to clear damaged mitochondria and prevent pyroptosis. Concurrently, ROS trigger antioxidant responses through the Keap1-Nrf2 pathway, regulating autophagy-related gene transcription to maintain cellular oxidative homeostasis. **(B)** Hypoxia: Under hypoxic conditions within the TME or deep within solid tumors, energy stress promotes the stable expression and nuclear translocation of HIF-1α, which upregulates BNIP3/NIX-related genes. By binding to LC3, it mediates the autophagic clearance of damaged mitochondria, reduces ROS, and maintains cellular energy homeostasis. **(C)** ER Stress: High protein synthesis demands readily trigger accumulation of unfolded proteins in the ER, activating the unfolded protein response (UPR). The PERK-eIF2α-ATF4 pathway transcritically activates autophagy-related genes such as LC3 and Atg12; The IRE1-JNK pathway, by phosphorylating Bcl-2, releases its inhibition on BECN1. This frees BECN1 to assemble active autophagy complexes, thereby buffering unfolded protein stress, delaying cell death, and aiding tumor cells in tolerating ER stress. **(D)** DNA Damage Stress: Platinum-based drugs such as cisplatin induce intrachromosomal/interchromosomal crosslinks and replication stress, activating the DNA damage response. Autophagy is induced and activated via molecules like ATG5 and ATG7, enhancing DNA repair efficiency and regulating cell cycle arrest. By degrading GATA4, autophagy suppresses cellular senescence processes, helping tumor cells cope with chemotherapy-induced DNA damage and contributing to the development of chemotherapy resistance.

## 3 The promoting role of autophagy in gynecologic malignant cells

### 3.1 Promoting effect of autophagy on survival of gynecologic malignant cells

Tumorigenesis and progression are often accompanied by adverse conditions including prolonged nutritional restriction, hypoxia, an acidic environment, and oxidative stress. Evidence indicates that gynecologic malignant cells are generally in a state of continuously activated autophagy, which plays a crucial protective role in maintaining their survival (Orfanelli et al., 2014). During nutrient deprivation, autophagy replenishes tumor cells with amino acids and fatty acids by degrading intracellular proteins and lipid droplets, thereby maintaining the essential substrate supply for mitochondrial oxidative phosphorylation and enabling tumor cells to sustain ATP production even under glucose-depleted or energy-deficient conditions (Thomas et al., 2018). In patient-derived ascites spheroid models, intact autophagy function is crucial for tumor cell survival in the nutrient-poor peritoneal environment. Inhibiting autophagy initiation significantly reduces spheroid viability and increases apoptosis (Singha et al., 2020). Autophagy-dependent survival mechanisms maysustain peritoneal free tumor cellsand are closely associated with disease recurrence, peritoneal dissemination, and poor clinical outcomes.

Beyond energy homeostasis, mitophagy significantly impacts treatment outcomes in gynecologic malignancies by enhancing mitochondrial quality. Cells of gynecologic malignancies are prone to mitochondrial damage under high ROS stress. Selective mitophagy promptly eliminates damaged mitochondria with impaired membrane potential, thereby halting sustained ROS release and maintaining mitochondrial homeostasis (Vara-Perez et al., 2019). By reducing mitochondrial-mediated oxidative damage and cell death signaling, tumor cells acquire a higher survival threshold under oxidative stress induced by platinum-based chemotherapy or radiotherapy, thereby diminishing the efficiency of treatment-induced apoptosis (Xia et al., 2025). At the same time, mitochondrial quality control contributes to stabilizing metabolic adaptability and proliferative potential, serving as a crucial foundation for the development of chemotherapy resistance and disease recurrence (Guan et al., 2021).

Under hypoxic conditions, HIF-1α induces the expression of BNIP3 and NIX. Both proteins directly bind LC3 through their LC3-interacting region (LIR) domains, triggering receptor-mediated mitophagy. This process eliminates damaged mitochondria generated during hypoxia, reduces ROS burden, and maintains basal energy supply (Wang S. et al., 2023). This mitophagypathway has been validated in ovarian and CC models, and is closely associated with tumor cell survival, adaptation to adverse environments, and the development of treatment resistance.

Across these contexts, autophagy serves as a critical protective mechanism that sustains energy availability, preserves mitochondrial quality, and maintains cellular homeostasis, thereby enabling tumor cells to adapt and survive under adverse conditions. In summary, autophagy establishes a sustained survival-promoting mechanism for gynecologic malignant cells by maintaining material and energy homeostasis, buffering oxidative stress, and ensuring mitochondrial quality. This chronically activated autophagic state not only propels tumors toward increased invasiveness and therapeutic resistance but also correlates with heightened recurrence risk, reduced progression-free survival, and diminished overall survival rates. Ultimately, it elevates disease-related mortality and significantly impacts the prognosis of gynecologic malignancies.

### 3.2 The role of autophagy in the TME

The microenvironment of gynecologic malignancies exhibits high complexity, characterized by hypoxia, chronic inflammation, and immunosuppression, accompanied by dynamic remodeling of various stromal cells such as fibroblasts and macrophages. Under this multifaceted stress context, tumor-cell-intrinsic autophagy regulates endogenous metabolism and stress adaptation, while autophagy-related processes in stromal and immune cells contribute to the dynamic remodeling of the TME, thereby influencing tumor progression (Zhang et al., 2021). On one hand, autophagy enhances the adaptability of gynecologic tumor cells to hypoxia, ROS accumulation, and inflammatory stimuli within the TME. In ovarian, cervical, and EC, these stress signals induce sustained autophagy activation. In turn, activated autophagy modulates the activity of inflammation-related pathways such as Interleukin-6 (IL-6)/Signal transducer and activator of transcription 3 (STAT3) and nuclear factor kappa-light-chain-enhancer of activated B cells (NF-κB), further supporting tumor cell survival and proliferation (Orfanelli et al., 2014; Liu et al., 2025). Simultaneously, autophagy helps maintain energy homeostasis and redox balance under hypoxic conditions, enabling tumor cells to sustain proliferative potential even under metabolically constrained environments. On the other hand, the interaction between autophagy and stromal cells serves as a key driver of microenvironment remodeling. Under metabolic or oxidative stress conditions, cancer-associated fibroblasts (CAFs) degrade intracellular components via autophagy, releasing metabolites such as lactate, amino acids, and ketone bodies to provide additional energy support for tumor cells—a phenomenon known as the reverse Warburg effect. This process is particularly pronounced in the OC microenvironment (Liang et al., 2022; Zhang et al., 2022).

Autophagy is also closely linked to the polarization of tumor-associated macrophages (TAMs). Within the TME, selective autophagy mediates cytoplasmic degradation of Nuclear factor NF-kappa-B(NF-κB) p65 subunit (p65), thereby reducing NF-κB activity and promoting the phenotypic shift of macrophages toward the M2 phenotype. Its molecular mechanism primarily relies on the autophagy receptor p62 recognizing and recruiting ubiquitinated p65, delivering it to the autophagolysosomal system for degradation, ultimately weakening pro-inflammatory and anti-tumor immune responses (Kuo et al., 2022; Li et al., 2023). Autophagy regulates TAMs functions including plasticity, polarization, antigen presentation, LC3-associated phagocytosis, and cytokine secretion, laying the foundation for their M2 polarization and tumor-promoting activities.

In gynecologic malignant TME, TAMs constitute one of the most abundant immune cell populations, predominantly exhibiting an M2-type immunosuppressive phenotype. High expression of markers such as Cluster of Differentiation 163, Cluster of Differentiation 206, and arginase one enables them to secrete pro-inflammatory and pro-angiogenic factors including IL-6, Transforming Growth Factor-beta (TGF-β), and vascular endothelial growth factor (VEGF),thereby enhancing tumor cell invasion, metastasis, and immune evasion, correlating closely with treatment resistance and poor prognosis (Krishnan et al., 2018; Jiang et al., 2019). For example, persistent infiltration of M2 TAMs in OC correlates positively with increased angiogenesis, matrix remodeling, and chemotherapy resistance (Schweer et al., 2022); In CC, HPV-driven chronic inflammation promotes TAMs polarization toward an immunosuppressive M2 phenotype, further weakening antiviral and antitumor immune responses (Chen and Zhao, 2025); A high proportion of M2 TAMs has also been observed in endometrial carcinoma tissues, where they promote tumor growth, angiogenesis, and metastasis by secreting VEGF, matrix metalloproteinases, and immunosuppressive chemokines (Sun et al., 2023). Additionally, metabolic byproducts such as lactic acid and fatty acids also participate in TAMs polarization, suggesting that metabolic interactions between tumors and macrophages play a significant role in gynecologic malignancies. Some studies further suggest that autophagy may enhance tumor immune evasion under certain conditions by regulating antigen presentation or maintaining Programmed Death-Ligand 1 (PD-L1) stability. However, direct evidence for this mechanism in gynecologic malignancies remains limited, with current insights primarily based on pan-cancer or *in vitro* model observations (Kuo et al., 2022).

In summary, autophagy is no longer merely a passive response of gynecologic tumor cells to adverse environments. Its effects on tumor cells and TME components are bidirectional and context-dependent, with tumor-cell-intrinsic autophagy supporting metabolic adaptation and survival under stress, while autophagy in stromal and immune cells may either promote or restrain tumor progression depending on the cellular and microenvironmental context.

### 3.3 The relationship between autophagy and chemotherapy resistance

Chemotherapy resistance is a major cause of treatment failure in gynecologic malignancies, particularly OC. Extensive research indicates that autophagy is a key mechanism by which tumor cells gain a survival advantage under chemotherapy stress (Niu et al., 2025). In platinum-based chemotherapy, autophagy promotes drug resistance through multiple pathways: On one hand, mitophagy clears chemotherapy-induced damaged mitochondria and oxidative stress products, reducing drug-induced apoptosis; On the other hand, autophagy blocks cytochrome C release by inhibiting the increase in mitochondrial outer membrane permeability, thereby lowering the threshold for caspase cascade activation. *In vitro* studies demonstrate that knockout of autophagy-related genes such as ATG7 and ATG14, or pharmacologically inhibiting autophagy, restores tumor cell sensitivity to platinum-based drugs (Yang et al., 2022; Wang and Wu, 2014). Furthermore, autophagy can further diminish the cytotoxic effects of chemotherapy-induced DNA damage by interacting with key tumor cell survival signaling pathways such as PI3K/Akt/mTOR and the DNA damage repair system (Pan et al., 2023; Roos et al., 2016). The mechanism may involve autophagy activation aiding tumor cells in maintaining the stability of DNA damage-sensing and repair-related proteins, thereby attenuating apoptotic signals and sustaining cell survival. This suggests that autophagy promotes drug resistance by jointly regulating signaling pathways and DNA repair (Zhang D. et al., 2015).

In OC, autophagy regulates the stability of DNA damage repair proteins such as RAD51, thereby influencing the efficiency of homologous recombination (HR) and tumor cell sensitivity to Poly ADP-ribose polymerase inhibitors (PARPi) (Ray et al., 2023). Additionally, autophagy may modulate responses to chemotherapy and PARPi by regulating the cyclic GMP-AMP synthase (cGAS)–Stimulator of interferon genes (STING) pathway. DNA damage or chromosomal instability can lead to cytoplasmic DNA accumulation, activating cGAS–STING-mediated innate immune signaling, inducing type I interferon responses, and enhancing antitumor immunity (Prabakaran et al., 2018; Ou et al., 2023). Selective autophagy can limit sustained activation of this pathway by clearing cytoplasmic DNA or degrading the cGAS/STING complex itself, thereby partially attenuating DNA damage-associated immune responses (Zheng et al., 2021).

Beyond platinum-based drugs, autophagy also modulates OC’s response to paclitaxel treatment. Paclitaxel exerts its antitumor effects by stabilizing microtubule structures, arresting cells in the Gap 2 phase/mitosis, and inducing mitotic catastrophe (Kampan et al., 2015). Research has revealed that paclitaxel induces a protectiveautophagic response in OC cells: on one hand, autophagy activation helps alleviate paclitaxel-induced ER stress and cell cycle arrest-related stress, reducing cell death signals triggered by mitotic abnormalities; on the other hand, autophagy promotes adaptive survival under drug stress by clearing abnormally aggregated tubulins and damaged organelles (Alalawy, 2024). Specifically, paclitaxel treatment induces upregulation of Thioredoxin Domain Containing 17 and BECN1 in OC cells, enhancing autophagic flux and conferring drug resistance. Conversely, inhibiting these autophagy-associated proteins or application of autophagy inhibitors significantly increases drug sensitivity and elevates apoptosis rates (Zhang S. et al., 2015); CREB regulated transcription coactivator 2 (CRTC2) enhances autophagy levels via the PI3K/Akt signaling pathway, reducing OC cells’ sensitivity to paclitaxel (Ou et al., 2023). Additionally, the intracellular stress induced by paclitaxel triggers protective autophagy. Inhibiting autophagy enhances paclitaxel’s cytotoxic effects, increases apoptosis rates, and restores drug sensitivity in certain drug-resistant models (Kubník et al., 2023).

Autophagy is also implicated in tumor tolerance to immunotherapy, with its potential mechanisms involving antigen presentation, major histocompatibility complex (MHC) class I molecule expression, and the regulation of stability in immune checkpoint proteins such as PD-L1 (Yamamoto et al., 2020). Studies using multiple tumor cell lines and co-culture models with immune cells indicate that autophagy activation reduces tumor antigen exposure, enhances immune evasion, and consequently diminishes tumor sensitivity to programmed cell death protein 1 (PD-1)/PD-L1 inhibitors. In OC, receptor xenotropic and polytropic retrovirus receptor 1 modulates major histocompatibility complex class I (MHC-I) molecule expression on tumor cell surfaces by regulating the autophagy pathway, thereby altering antigen presentation capacity and influencing immune recognition and tumor growth (Wang et al., 2024). In endometrial carcinoma, autophagy suppresses NLR family CARD domain containing 5 (NLRC5) expression via an LC3-dependent mechanism, thereby reducing MHC-I gene transcription and antigen presentation, ultimately promoting immune evasion (Zhan et al., 2022). Furthermore, analysis of autophagy-related gene signatures in CC suggests that autophagy status may be closely associated with the tumor immune microenvironment and prognosis (Li et al., 2024).

In summary, autophagy represents a crucial adaptive mechanism through which gynecologic malignancies acquire resistance under various therapeutic pressures. Its role extends beyond a single drug class, instead systematically attenuating treatment-induced cytotoxic effects via multi-tiered pathways. Currently, several critical gaps in autophagy-mediated drug resistance research limit its clinical translational value. At the DNA damage repair level, although studies suggest that autophagy stabilizes RAD51 and impacts HR repair efficiency, direct mechanistic evidence remains lacking regarding whether autophagy selectively degrades HR inhibitors or directly regulates the breast cancer type 1 susceptibility protein (BRCA1)–RAD51 assembly process. Identifying the specific molecular targets through which autophagy preferentially regulates HR versus non-homologous end-joining repair pathways could help explain the differential sensitivity of different OC subtypes to platinum-based drugs and PARPi. The various tumor-promoting effects and mechanisms of autophagy in gynecologic malignancies are systematically summarized in Figure 3.

**FIGURE 3:**
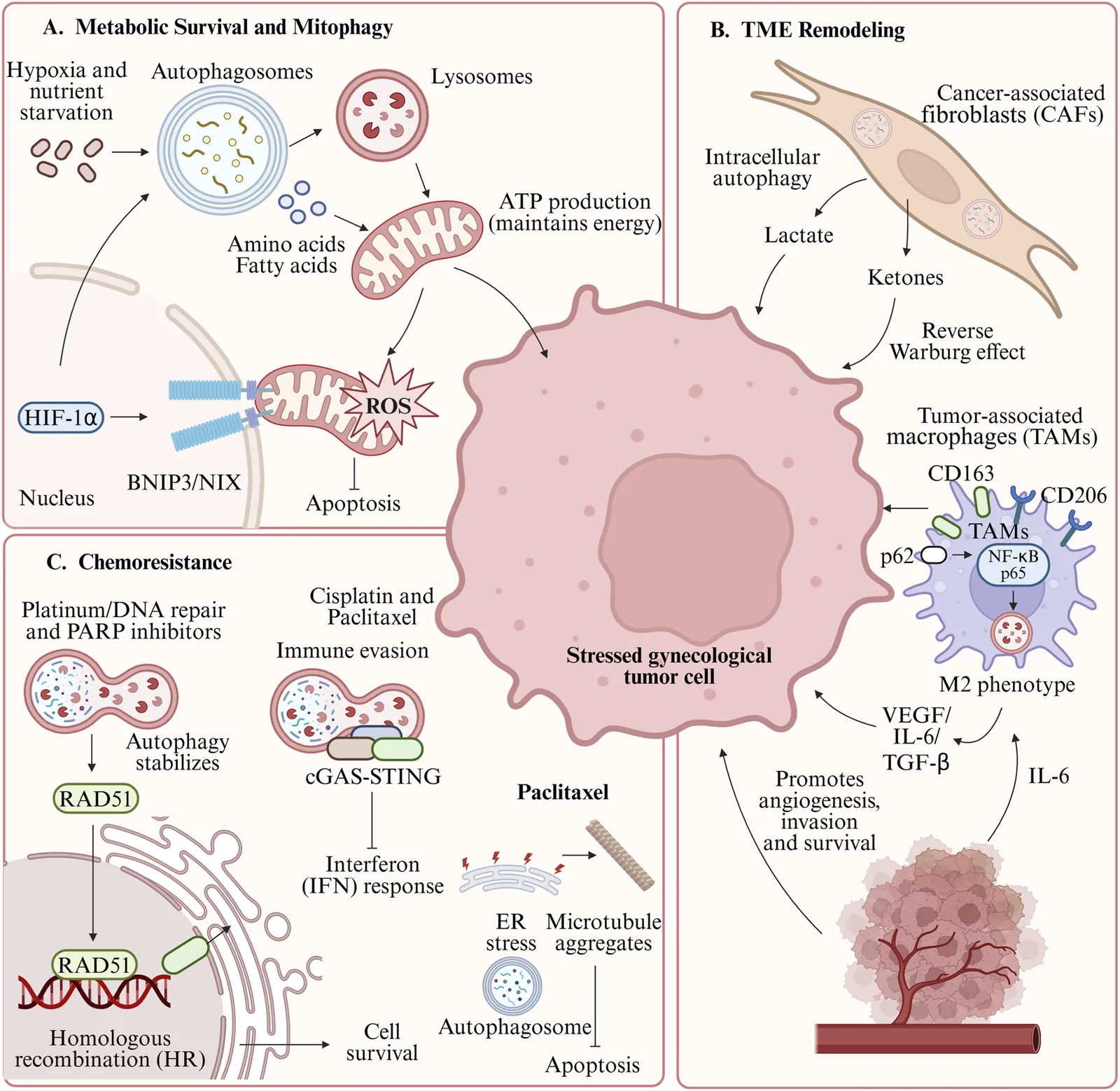
The Promoting Role of Autophagy in Gynecologic Malignant Tumors. **(A)** Metabolic Survival and Mitochondrial Autophagy: Under hypoxia or nutrient deprivation, the HIF-1α pathway is activated, upregulating BNIP3/NIX to induce LC3-mediated mitochondrial autophagy. This process eliminates damaged mitochondria, reduces reactive oxygen species (ROS), and maintains ATP production, thereby suppressing apoptosis and promoting tumor cell survival in adverse microenvironments. **(B)** TME Remodeling: Tumor cells induce intracellular autophagy in CAFs, which release metabolic byproducts such as lactic acid and ketone bodies. Through the “reverse Warburg effect,” these substances provide energy substrates to tumor cells. Interactions with TAMs: Tumor cells and M2-type TAMs secrete cytokines such as IL-6 and VEGF, promoting angiogenesis to supply nutrients and oxygen for tumor growth. Concurrently, signaling molecules like NF-κB and p62 within TAMs further regulate inflammatory and immunosuppressive states, enhancing tumor cell survival and progression. **(C)** Chemotherapy Resistance and Immune Evasion: Autophagy enhances homologous recombination (HR) repair capacity by stabilizing RAD51 protein, efficiently repairing DNA damage caused by platinum-based chemotherapeutics (e.g., cisplatin), thereby mediating tumor cell resistance to chemotherapy. It activates the cGAS-STING pathway, suppresses the interferon (IFN) response, and weakens anti-tumor immune surveillance. Simultaneously, autophagy clears taxol-induced ER stress aggregates and microtubule damage, preventing apoptosis and ultimately promoting tumor cell survival and immune evasion under therapeutic stress.

## 4 The inhibitory role of autophagy in gynecologic malignancies

### 4.1 Mechanisms of autophagy in tumor development suppression

Autophagy plays a crucial tumor-suppressing role in the early stages of gynecologic malignancies, with its core functions being the maintenance of cellular quality control, the limitation of gene mutations, and the mitigation of carcinogenic effects driven by chronic inflammation. First, autophagy prevents cells from entering a state of genomic instability by selectively eliminating damaged mitochondria and misfolded proteins, thereby reducing ROS production and DNA double-strand breaks (Mathew et al., 2009; Vessoni et al., 2013). In cervical intraepithelial neoplasia (CIN), HPV E6/E7 proteins induce mitochondrial accumulation and impaired autophagy by suppressing the PTEN-induced kinase 1/parkin pathway, thereby exacerbating oxidative damage and promoting abnormal proliferation of the epithelial basal layer (Lagunas-Martínez et al., 2022; Belleudi et al., 2015). Research indicates that as CIN grades increase, the expression of autophagy-related regulatory factors such as BECN1, LC3, ER membrane protein complex subunit 6 (EMC6), and Ras-related protein Rab-5A(Rab5A) progressively declines. This suggests that autophagy defects may drive the development of CC, while restoring autophagy activity may help slow CIN progression (Hu et al., 2015; Ma et al., 2022; Shen and Ding, 2017).

Second, in endometrial carcinoma, dysregulated autophagy is closely associated with abnormalities in key tumor suppressor genes. Loss of AT-rich interaction domain 1A (ARID1A) impairs SWItch/sucrose non-fermentable (SWI/SNF)-mediated chromatin remodeling, disrupting transcription of multiple stress and homeostasis-related genes—including core autophagy factors such as ULK1 and ATG5—thereby reducing autophagic flux. This phenomenon has been consistently observed in both ARID1A-deficient endometrial carcinoma and ovarian clear cell carcinoma (Toumpeki et al., 2019; Suryo et al., 2020). ARID1A deficiency not only disrupts cellular protein homeostasis but also impairs multiple pathways including DNA damage repair, metabolic regulation, and autophagy modulation, driving the evolution of endometrial carcinoma toward a more invasive phenotype (Toumpeki et al., 2019). Furthermore, studies have confirmed that ARID1A deficiency relieves HDAC-mediated repression of inflammatory genes while enhancing NF-κB signaling and inducing the expression of multiple inflammatory cytokines. This creates a pro tumorigenic chronic inflammatory microenvironment. These changes synergistically interacts with the accumulation of cellular stress caused by impaired autophagy, jointly promoting tumor progression (Kim et al., 2016). Third, Serous tubal intraepithelial carcinoma (STIC) represents the direct precursor lesion to HGSOC. Tumor Protein 53 (TP53) inactivation is a common molecular event in this lesion, and loss of TP53 function directly impairs autophagy initiation (Kraiczy and Yu, 2025). Studies using clinical samples and STIC models indicate that reduced autophagy levels render fallopian tube epithelial cells more susceptible to DNA damage accumulation and chromosomal instability, thereby promoting the development of high-grade serous carcinoma. Conversely, enhancing autophagy reduces the transformation potential of these precancerous cells (Bronder et al., 2021).

In summary, autophagy slows the onset and early progression of gynecologic malignancies by eliminating harmful intracellular structures, maintaining energy and protein homeostasis, and limiting chronic inflammatory responses. Its tumor-suppressing effects are particularly pronounced in HPV-associated precancerous lesions, ARID1A-deficient cancers, and TP53-driven OC development.

### 4.2 The inhibitory role of autophagy in the metastasis and dissemination of gynecologic malignancies

The metastatic patterns of gynecologic malignancies exhibit distinct tissue specificity: OC primarily spreads via peritoneal dissemination, CC mainly involves local invasion and lymphatic metastasis, EC manifests as adenomyotic infiltration and distant metastasis, while vulvar and vaginal cancers tend toward local expansion and lymphatic spread (Hoivik and Sergi, 2022). Despite differences in metastatic patterns, autophagy consistently inhibits metastasis across various gynecologic malignancies through multi-tiered mechanisms that restrict tumor spread. These include suppressing epithelial-mesenchymal transition (EMT), regulating cell adhesion, inhibiting matrix degradation, and modulating the microenvironment. First, autophagy selectively degrades key EMT transcription factors to inhibit epithelial-to-mesenchymal conversion, thereby maintaining epithelial phenotype stability (Gundamaraju et al., 2022). Autophagy activation is closely associated with the maintenance of epithelial cadherin (E-cadherin) expression, attenuation of the EMT phenotype, and reduced tumor cell migration capacity. It may limit tumor cell invasion by inhibiting EMT-related cellular structural and dynamic alterations. Conversely, impaired autophagy leads to abnormal upregulation of EMT drivers, disruption of intercellular junctions, and significantly enhanced tumor invasiveness. In HPV-associated CC, inhibiting Sorting nexin-10 induces autophagy activation by suppressing PI3K/AKT signaling, significantly reversing the EMT phenotype and reducing cell invasiveness. This suggests a direct causal relationship between autophagy deficiency and sustained EMT activation (Liao et al., 2025). In OC models, Mitofusin 2 (MFN)-mediated mitochondrial fusion suppresses EMT progression and tumor invasion by reducing ROS levels and promoting autophagy activation through regulation of the AMPK/mTOR/ERK signaling pathway. This further reveals the tumor-suppressing role of the autophagy-EMT axis in gynecologic malignancies at the mitochondrial homeostasis level (Ashr et al., 2022).

Second, peritoneal dissemination in OC is initiated by tumor cell detachment and relies on the integrin–focal adhesion kinase (FAK) axis for adhesion to the peritoneal mesothelium and subsequent transmesothelial migration. Integrins bind to the extracellular matrix, activating FAK and triggering downstream signaling pathways that enhance cell adhesion, migration, and metastatic potential. This mechanism plays a central role in the peritoneal spread of high-grade OC. (Dhaliwal and Shepherd, 2022). As a system for maintaining intracellular homeostasis, autophagy is also closely linked to the dynamic regulation of adhesion complexes. For instance, autophagy promotes the disassembly of focal adhesion structures by interacting with focal adhesion proteins such as paxillin via LC3, thereby influencing the migration and invasion capabilities of tumor cells (Sharifi et al., 2016). Autophagy may reduce tumor cell adhesion and peritoneal seeding efficiency by disrupting the homeostasis of the focal adhesion complex and inhibiting the sustained activation of integrin-FAK signaling. Furthermore, autophagy participates in tumor dissemination by regulating the TME, such as influencing the secretory function of tumor-associated fibroblasts, modulating the survival and functional state of TAMs, and maintaining vascular and lymphatic endothelial homeostasis. In the ID8 mouse model of OC peritoneal metastasis, TAMs exhibiting high oxidative phosphorylation capacity demonstrate elevated mitophagy activity. Interfering with their autophagy process impairs macrophage survival and enhances antitumor immune responses, thereby significantly suppressing peritoneal metastasis (Xia et al., 2020).

Of note, that autophagy exhibits distinct stage-specific and context-dependent functions in gynecologic malignancies: In the early stages of tumorigenesis or primary tumor growth, tumor cells primarily confront nutrient deprivation, hypoxia, and treatment-related stress. Autophagy significantly enhances tumor cell survival and treatment tolerance by maintaining energy homeostasis, mitochondrial quality, and protein homeostasis. However, as tumors progress to invasion and metastasis, cellular migration, adhesion remodeling, and EMT become decisive events for tumor dissemination. At this stage, excessive or sustained autophagy activation can instead inhibit tumor spread by restricting the EMT program, disrupting adhesion complex homeostasis, and reshaping the metastatic microenvironment. Overall, autophagy does not play a passive role; rather, it exerts systemic constraints throughout the entire process of tumor invasion, detachment, dissemination, and re-implantation through multidimensional regulation of both the cell itself and its microenvironment.

### 4.3 Promoting and inhibitory roles of autophagy in immunoregulation of gynecologic malignancies

Autophagy not only maintains intracellular homeostasis but also participates in the processing of tumor antigens and MHC-I-restricted presentation, influencing tumor cell recognition by CD8-positive T lymphocytes (CD8^+^ T cells). It similarly exhibits bidirectional effects in the immune regulation of gynecologic malignancies and is closely associated with tumor molecular subtypes. Autophagy plays a crucial role in regulating MHC class I molecule presentation of endogenous antigens, enhancing tumor antigen display on the cell surface and promoting CD8^+^ T cell-mediated immune surveillance (Cr et al., 2009; Ynebr, 2020). In HPV-positive CC, the oncogenic HPV E6/E7 proteins not only drive cellular transformation but also participate in immune evasion mechanisms by influencing antigen processing and presentation pathways, thereby suppressing host antiviral immunity. This represents a key mechanism underlying HPV persistent infection and immune tolerance (Senba and Mori, 2012). Based on this, enhancing autophagy activity to increase the entry of HPV E6/E7 antigens into the MHC-I processing pathway and their recognition by CD8^+^ T cells is considered a potential strategy for boosting the immune response against HPV-positive tumors.

Autophagy also participates in the regulation of immune checkpoint proteins. PD-L1 protein can be degraded via the lysosomal/autophagic pathway, reducing PD-L1 expression on tumor cell surfaces. This relieves the inhibition of CD8^+^ T cells, enhances antitumor immunity, and increases tumor cell sensitivity to PD-1/PD-L1 inhibitors. For instance, PD-L1 can undergo autophagic degradation mediated by huntingtin-interacting protein 1-related (HIPIR), thereby enhancing T cell–mediated cytotoxicity and suggesting that this mechanism may sensitize tumors to immunotherapy. CMA has also been demonstrated to reduce PD-L1 expression, further supporting autophagy’s negative regulation of PD-L1 through multiple pathways (Cui et al., 2023), This regulatory mechanism holds particular significance for the efficacy of immune checkpoint inhibitors in tumor types with pronounced immune evasion, such as OC. In endometrial carcinoma, autophagy exerts subtype-specific effects on immune regulation: autophagy protein LC3 directly interacts with NLRC5, inhibiting NLRC5-driven MHC-I antigen presentation, thereby reducing tumor visibility to CD8^+^ T cells and diminishing their cytotoxic capacity (Zhan et al., 2022). Simultaneously, autophagy exhibits intricate interactions with the cGAS-STING and type I interferon pathways. Activation of cGAS-STING can induce type I interferon production, promote antigen presentation and dendritic cell maturation, and enhance antitumor immunity (Lu et al., 2023).

In endometrial carcinoma subtypes with high mutation burdens, such as microsatellite instability-high (MSI-H) and DNA polymerase epsilon (POLE), catalytic subunit mutant, elevated tumor neoantigen load and significant immune infiltration are observed. Enhanced autophagy may amplify antigen presentation and immune activation pathways, thereby improving tumor response to immune checkpoint inhibitors (Dessources et al., 2023).

In OC with dominant TP53 mutation, the immune evasion mechanisms driven by TP53 mutations are complex, encompassing the establishment of an immunosuppressive microenvironment and suppression of antigen presentation. Under these conditions, autophagy tends to support immune evasion rather than enhance immune killing (Silwal-Pandit et al., 2017). Therefore, autophagy exhibits distinct molecular subtype specificity in the immunoregulation of gynecologic tumors: in certain subtypes, enhanced autophagy is crucial for improving immunotherapy efficacy; whereas in other subtypes, inhibiting autophagy may be more beneficial for promoting antitumor immune responses. The core tumor-suppressive role of autophagy in gynecologic malignancies and its molecular mechanisms are comprehensively depicted in Figure 4.

**FIGURE 4:**
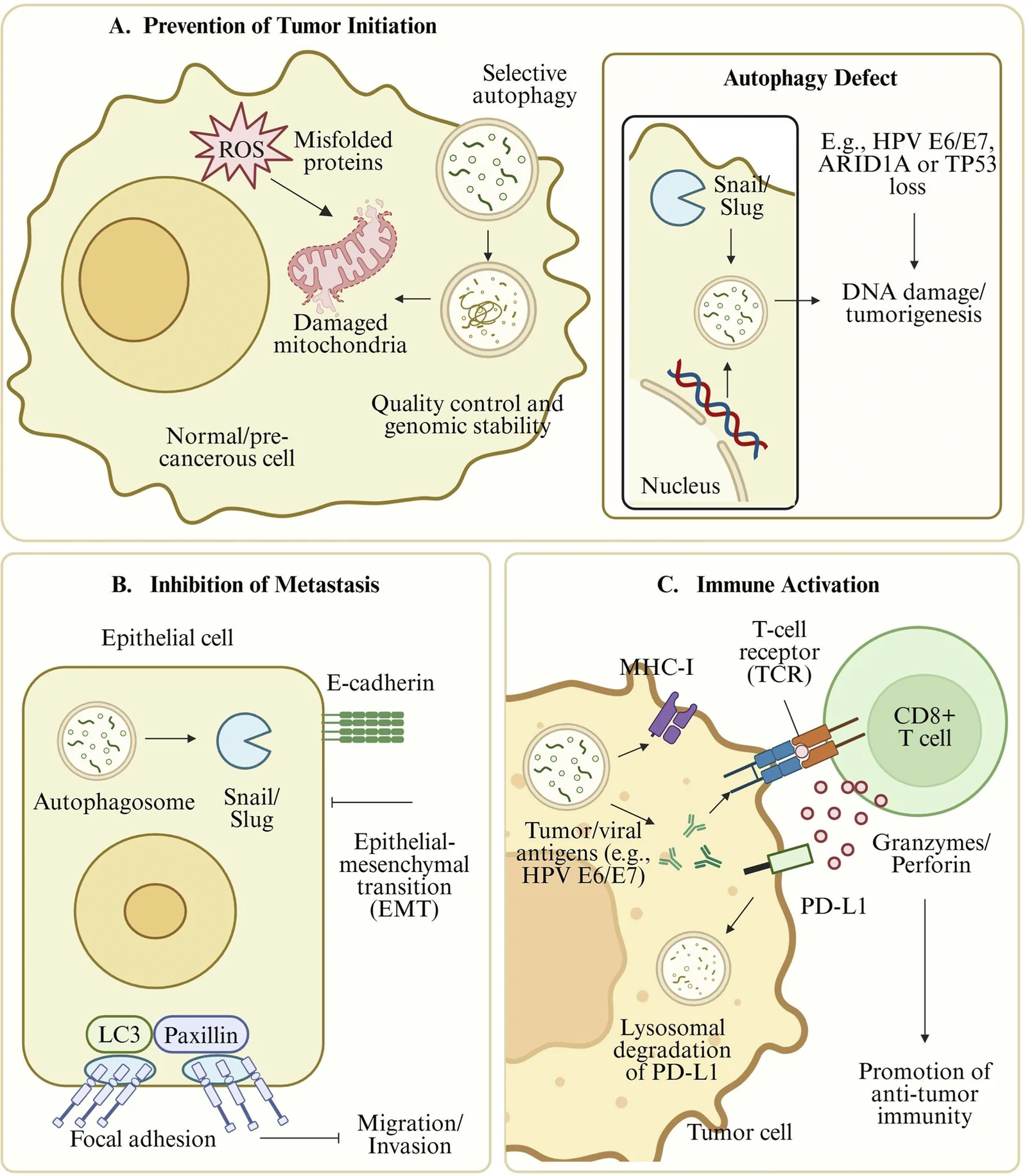
The Inhibitory Role of Autophagy in Gynecologic Malignant Tumors. **(A)** In the early stages of tumorigenesis, autophagy prevents precancerous lesions from progressing to malignancy by clearing damaged mitochondria, misfolded proteins, and reactive oxygen species (ROS), thereby maintaining genomic stability and cellular quality control. When autophagy is impaired due to HPV E6/E7 or ARID1A loss, DNA damage and inflammation accumulate, accelerating tumor development. **(B)** During the metastasis stage, autophagy suppresses cell migration and invasion by degrading EMT drivers such as Snail/Slug, maintaining E-cadherin expression, and regulating focal adhesion complex stability, thereby limiting tumor dissemination. **(C)** At the immune level, autophagy promotes MHC-I presentation of tumor antigens and degrades PD-L1 via the lysosomal pathway, thereby enhancing CD8^+^ T-cell killing activity.

## 5 Regulatory factors of autophagy in gynecologic malignancies

### 5.1 Effects of gene mutations on autophagy

In gynecologic malignancies, multiple gene mutations can directly or indirectly affect autophagy activity, thereby participating in tumorigenesis, progression, and treatment response regulation. Among these, tumor suppressor genes associated with the PI3K/Akt/mTOR signaling axis (e.g., ARID1A, PTEN) primarily regulate autophagy initiation and flux by influencing signal transduction or chromatin remodeling states; mutations in core cellular stress and homeostasis factors (e.g., TP53) can disrupt autophagic homeostasis and promote genomic instability; DNA damage repair-related genes (e.g., BRCA1/2) often undergo adaptive activation of DNA damage-associated autophagy in the context of HR defects. This article systematically reviews the mutation characteristics and molecular mechanisms of autophagy regulation for these representative genes.

#### 5.1.1 Mutations in tumor suppressor genes associated with the PI3K/Akt/mTOR axis: ARID1A and PTEN

ARID1A and PTEN are the most representative autophagy-regulating tumor suppressor genes in gynecologic malignancies. Both can influence autophagic flux through the PI3K/Akt/mTOR signaling axis, yet their functional hierarchy and regulatory mechanisms exhibit significant differences.

ARID1A is a core tumor suppressor gene of the SWI/SNF chromatin remodeling complex. Its loss is not only closely associated with the development and malignant progression of gynecologic malignancies, but also suppresses autophagic flux by activating the PI3K/Akt/mTOR signaling axis (Bosse et al., 2013). In ARID1A knockout cell models, enhanced activity of the PI3K/Akt/mTOR pathway and decreased expression of autophagy-related proteins suggest that ARID1A deficiency modifies autophagy-related gene expression through upstream signaling pathways, thereby inhibiting autophagy activity (Sun et al., 2022). ARID1A loss frequently occurs in ovarian clear cell carcinoma and endometrial carcinoma, correlating with genomic instability and malignant progression phenotypes, suggesting its crucial regulatory role in downstream homeostasis pathways including autophagy (Suryo et al., 2020).

PTEN serves as a key negative regulator in the PI3K/Akt/mTOR signaling axis. Its mutations or loss are highly prevalent in gynecologic malignancies and are closely associated with autophagy regulation. PTEN inhibits PI3K/Akt signaling by dephosphorylating Phosphatidylinositol (Amant et al., 2005; Stewart et al., 2019; Konstantinopoulos and Matulonis, 2023)-trisphosphate (PIP3), thereby indirectly suppressing mTORC1 activity and maintaining basal autophagy levels. Loss of PTEN function leads to sustained activation of the PI3K/Akt/mTOR pathway, inhibition of the ULK1 complex, and impaired autophagy initiation, ultimately weakening cellular responses to metabolic stress and the clearance of damaged organelles (Peng et al., 2022). In endometrial carcinoma, PTEN mutations predominantly occur in the early stages of the disease and represent a key driver event in tumorigenesis. The concomitant inhibition of autophagy may promote genomic instability and tumor initiation (Fukuda and Wada-Hiraike, 2022). Meanwhile, residual autophagy activity in tumor tissues with PTEN loss may be repurposed by tumor cells to adapt to nutrient-deprived and hypoxic microenvironments (Peng et al., 2016). Unlike ARID1A, PTEN exerts its influence on autophagy primarily through switch-like regulation at the signal transduction level, producing effects that are more rapid and highly coupled with cellular nutrition and energy status.

#### 5.1.2 TP53 mutations and autophagy homeostasis Imbalance

Similarly, in HGSOC and its precursor lesions, TP53 inactivation is one of the most common driver events (Silwal-Pandit et al., 2017). Wild-type p53 plays a crucial role in regulating autophagy by transcribing autophagy-related genes and maintaining clearance functions under cellular stress. TP53 mutations not only lose these regulatory functions but also indirectly impede autophagy initiation by disrupting cellular homeostasis networks. This leads to the accumulation of damaged mitochondria and DNA damage, thereby increasing the risk of tumorigenesis (Rahman et al., 2022). Furthermore, oncogenic signaling pathways such as PI3K/Akt/mTOR are frequently persistently activated in endometrial and OC. By activating mTORC1, they inhibit the ULK1 complex and downstream autophagy execution programs, thereby aiding tumor cells in evading immune surveillance and cell death (Rosenfeldt and Ryan, 2009; Zhang et al., 2023).

#### 5.1.3 BRCA1/2 mutations

BRCA1/2 mutations represent a key molecular hallmark of OC and certain EC. Their core pathological basis lies in HR deficiency, leading to persistent accumulation of DNA double-strand breaks and heightened chromosomal instability. Against the backdrop of BRCA1/2 deficiency, HR repair dysfunction in tumor cells leads to increased accumulation of DNA damage, often accompanied by the activation of DNA damage-associated autophagy. This represents a crucial adaptive mechanism for tumor cells responding to the stress of platinum-based chemotherapy and PARPi (Santiago-O’Far et al., 2020). BRCA1-deficient tumors can also activate the STING signaling axis through cytoplasmic DNA accumulation, thereby influencing the tumor immune microenvironment and treatment response (Bruand et al., 2021). Furthermore, the regulatory role of BRCA genes extends to the molecular level of autophagy-immune interactions, potentially influencing treatment-related immune responses and antitumor activity within tissues (Ding et al., 2018; Morand et al., 2020).

It is important to note that the impact of gene mutations on autophagy is not consistent across all tumor types or stages: in precancerous lesions or early-stage tumors, impaired autophagy may promote genomic instability and tumorigenesis; whereas in established tumors, partially suppressed autophagy can confer a survival advantage to cancer cells under metabolic stress. Furthermore, autophagy frequently intertwines with processes such as apoptosis, senescence, and immune evasion, further highlighting its multi-layered regulatory role within the TME. The specific effects of different gene mutations on autophagy regulation, key signaling pathways, and biological significance are comprehensively summarized in Table 1.

**TABLE 1:** Effects of gene mutations on autophagy regulation in gynecologic malignancies.

Gene	Mutational characteristics and occurrence context	Key signaling pathways and mechanisms	Effects on autophagy	Biological and therapeutic significance	References
ARID1A	OC and EC Medium-to-High Frequency Inactivation	Chromatin remodeling defects → Enhanced PI3K/Akt/mTOR signaling	Inhibition of ULK1 → Autophagy initiation and decreased flux	Promoting genomic instability and tumor progression	(Suryo et al., 2020; Bosse et al., 2013; Sun et al., 2022)
PTEN	Common Early-Stage Deficiencies in EC	PI3K/Akt sustained activation → enhanced mTORC1 activity	Autophagy initiation is impaired	Promote tumorigenesis; late-stage residual autophagy supports metabolic adaptation	(Peng et al., 2022; Fukuda and Wada-Hiraike, 2022; Peng et al., 2016)
TP53(WT)	Tumor suppressor gene retention expression	Transcription activation of autophagy genes such as DRAM1	Promoting autophagy under stress conditions	Maintain genomic stability and suppress tumorigenesis	(Ryan, 2011; Rahman et al., 2022)
TP53(Mut)	HGSOC High-to-Mid-Frequency Sudden Change	Loss of transcription function; disruption of stress signaling	Autophagy homeostasis imbalance	Accumulation of DNA Damage and Chromosomal Instability	(Silwal-Pandit et al., 2017; Rahman et al., 2022)
BRCA1/2	HR defects in OC and some EC	DNA damage continues to accumulate → STING activation	DNA damage-associated autophagy enhancement	Promoting Adaptive Resistance to PARPi and Platinum-Based Therapies	(Santiago-O’Far et al., 2020; Bruand et al., 2021; Ding et al., 2018; Morand et al., 2020)

### 5.2 Mechanisms of drug effects on autophagy

Drug intervention serves as a core exogenous factor regulating autophagy activity in gynecologic malignancies, exhibiting distinct “double-edged sword” characteristics: certain anticancer therapies induce protective autophagy, enhancing tumor cells’ tolerance to therapeutic stress; conversely, inhibiting key autophagy pathways weakens tumor cells’ adaptive capacity, increasing treatment sensitivity. Consequently, the functional role of autophagy in drug responses depends on tumor type, molecular context, and treatment stage. Traditional chemotherapeutic agents activate autophagy in tumor cells by inducing DNA damage, mitochondrial dysfunction, and ROS accumulation. As a protective response, autophagy helps tumor cells clear damaged mitochondria and misfolded proteins while maintaining energy supply, thereby reducing chemotherapy-induced cell death and promoting drug resistance development (Li et al., 2017; Chavez-Dominguez et al., 2020). Classic platinum-based drugs such as cisplatin can significantly induce autophagy in multiple tumor cell models, upregulate autophagy-related markers, and participate in adaptive regulation of ROS and DNA damage. This process is typically associated with reduced sensitivity of tumor cells to chemotherapy (Xu and Gewirtz, 2022). Additionally, the development of chemotherapy resistance is associated with the enhancement of protective pathways such as mitophagy. These pathways reduce ROS burden by removing damaged mitochondria and maintain cellular homeostasis, thereby increasing tumor cell resistance (Bahar et al., 2022).

Given autophagy’s protective role in the development of chemotherapy resistance, autophagy inhibitors are widely explored as potential strategies to reverse tumor drug resistance. Among these, chloroquine (CQ) and hydroxychloroquine (HCQ) are the most extensively studied autophagy-inhibiting drugs. As classic lysosomal inhibitors, both drugs elevate lysosomal pH, thereby blocking the fusion of autophagosomes with lysosomes. This leads to the abnormal intracellular accumulation of autophagic substrates and damaged organelles, amplifying oxidative stress and mitochondrial dysfunction, and ultimately inducing cytotoxic responses (Zhu et al., 2017; Fukuda et al., 2015). However, the application of CQ/HCQ in gynecologic malignancy models presents significant limitations: First, ovarian and EC often exhibit high tumor burden, peritoneal dissemination, or dense tumor stroma. CQ/HCQ demonstrates limited drug penetration and lysosomal accumulation efficiency within solid tumor tissues, making it difficult to achieve sustained, sufficient inhibition of autophagic flux at clinically tolerable doses (Abdel Karim et al., 2025). Second, the precise mechanism of autophagy inhibition by CQ/HCQ and its clinical response remain incompletely understood. Its efficacy shows inconsistencies across different tumor contexts, and stable autophagy-inhibitory effects are difficult to achieve when used as monotherapy (Ferreira et al., 2021). Third, HCQ’s retinal toxicity and other dose-dependent side effects also limit its widespread clinical use in combination therapy (Shi et al., 2017).

Molecularly targeted drugs primarily regulate autophagy by influencing the PI3K/Akt/mTOR signaling axis. As a key negative regulator at the initiation stage of autophagy, mTOR enhances mTORC1 activity under conditions of nutrient sufficiency or growth factor stimulation. This activation phosphorylates and inhibits the ULK1 complex, thereby blocking autophagy initiation. Conversely, when mTOR signaling is suppressed, the ULK1 complex becomes activated, initiating the autophagic process. Notably, in gynecologic malignancies, autophagy induced by molecular targeted drugs exhibits distinct dual biological effects: excessive or sustained autophagy inhibits tumor growth through energy depletion or organelle damage; conversely, under therapeutic stress, autophagy may function as an adaptive stress response, providing metabolic buffering and survival advantages to tumor cells, thereby diminishing targeted therapy efficacy. Rapamycin and its derivatives (rapamycin analogs) specifically inhibit mTORC1 activity, serving as classic autophagy inducers that significantly enhance autophagy initiation levels across multiple gynecologic malignancy models (Rezazadeh et al., 2020; Eum et al., 2011). PI3K or Akt inhibitors can also indirectly relieve mTOR’s inhibition of autophagy by blocking upstream activating signals of mTOR, thereby promoting autophagy.

Beyond targeted agents for the PI3K/Akt/mTOR signaling axis, clinically widely used PARPi indirectly regulate the autophagy pathway via DNA damage stress. Their effects in gynecologic malignancies exhibit marked tumor type and molecular background dependence, particularly prominent in HGSOC and certain EC. HGSOC demonstrates a high incidence of HR deficiency (HRD), PARPi exert significant cytotoxicity by exacerbating DNA damage burden. Concurrently, they induce LC3-II accumulation, BECN1 upregulation, and enhanced autophagic flux, suggesting that autophagy is activated as a stress adaptation mechanism. During the phase of acquired drug resistance, autophagy supports tumor cell survival by clearing debris from DNA damage-related organelles, buffering replication stress, and maintaining energy homeostasis (Apelian et al., 2025). In PTEN-deficient endometrial carcinoma cell models, activation of the PI3K/mTOR pathway is closely associated with PARPi sensitivity, suggesting that the signaling pathway context is a critical factor influencing the autophagic response of tumor cells to PARPi (Bian et al., 2018; Philip et al., 2017). The aforementioned discrepancies indicate that autophagy’s role in PARPi therapy for gynecologic malignancies is not unidirectional but rather multifactorially regulated by tumor histological type, HRD status, PI3K pathway activity, and other factors.

Antibody-drug conjugates (ADCs) have demonstrated potential therapeutic activity in gynecologic malignancies as an emerging targeted treatment modality. They primarily target tumor-associated antigens such as folate receptor alpha (FRα) and trophoblast cell surface antigen 2, relying on the endocytosis–endosome/lysosome pathway to release their payload and exert their effects. The intracellular fate of ADCs is closely linked to the autophagy status of tumor cells (Lee and Liu, 2019). Treatment with ADCs induces protective autophagy responses, while inhibiting autophagy enhances the cytotoxic effects of ADCs, suggesting that the autophagy state may regulate tumor cell response to ADC therapy (Xue et al., 2024). In OC models, FRα-targeted ADCs not only exert payload toxicity but also enhance autophagy marker levels, further confirming autophagy’s involvement in ADC effect modulation (Chelariu-Raic et al., 2025).

Additionally, the regulatory role of autophagy in immunotherapy-related drugs has garnered increasing attention: autophagy participates in tumor antigen processing, MHC-I presentation, and the regulation of immune checkpoint protein stability. Some studies suggest that enhanced autophagy promotes PD-L1 degradation via the lysosomal pathway, reducing PD-L1 expression on tumor cell surfaces and enhancing CD8^+^ T cell-mediated immune killing. Conversely, under different circumstances, autophagy may also support immune evasion by maintaining tumor cell homeostasis. This dual role underscores the critical importance of precisely regulating autophagy within immunotherapy combination regimens.

Overall, in gynecologic malignancies, autophagy functions both as a buffering mechanism against therapeutic-induced stress—promoting cell survival and drug resistance—and as a participant in cell death execution under specific conditions. This dual role suggests that whether autophagy constitutes an effective therapeutic intervention target depends not on its activation or inhibition, but rather on the tumor’s molecular context, the stage of treatment, and the dynamic changes in autophagic flux. To summarize these processes clearly Table 2 outlines the main drug categories, how they modulate autophagy, and the resulting biological effects.

**TABLE 2:** Drug regulation of autophagy and its significance in the treatment of gynecologic malignancies.

Drug category	Representative drugs	Autophagy regulatory mechanisms	The role of autophagy in this context	Significance of treatment strategies	References
Platinum-based chemotherapy	Cisplatin, Carboplatin	DNA damage and ROS accumulation → AMPK activation → enhanced a autophagic flux; Induction of mitochondrial autophagy	Primarily protective autophagy, clearing damaged mitochondria and maintaining ATP supply	Promotes drug resistance formation; inhibiting autophagy enhances chemotherapy sensitivity	(Li et al., 2017; Chavez-Dominguez et al., 2020; Xu and Gewirtz, 2022; Bahar et al., 2022)
Taxaceae	Paclitaxel	Mitotic stress and ER stress → autophagy activation	Buffering microtubule abnormalities and stress damage, reducing apoptosis	Inhibition of autophagy enhances cytotoxicity	(Kampan et al., 2015; Alalawy, 2024; Zhang et al., 2015b; Ou et al., 2023; Kubník et al., 2023; Yamamoto et al., 2020; Wang et al., 2024; Zhan et al., 2022; Li et al., 2024; Mathew et al., 2009)
Lysosomal autophagy inhibitor	CQ, HCQ	Elevating lysosomal pH → Inhibits autophagosome-lysosome fusion → Impedes autophagic flux	Blocking protective autophagy enhances ROS and apoptosis	Combination chemotherapy can reverse drug resistance, but its clinical adoption is limited by toxicity concerns	(Zhu et al., 2017; Fukuda et al., 2015; Abdel Karim et al., 2025; Ferreira et al., 2021; Shi et al., 2017; Xu et al., 2018; Pagotto et al., 2017; Hwang et al., 2020; Nu Ez-Olvera et al., 2019)
Novel autophagy inhibitor	Lys05, Bafilomycin A1	Inhibition of lysosomal acidification or V-ATPase function → Blocking of autophagic flux	More potent inhibition of autophagy-dependent survival	High potential for preclinical combination therapy	(Amaravadi and Winkler, 2012; Rebecca et al., 2019; Wang et al., 2021)
mTOR inhibitors (autophagy inducers)	Rapamycin, Rapalogs	Inhibition of mTORC1 → Activation of ULK1 → Enhancement of autophagy initiation	Context-dependent: Can induce either “burden autophagy” or cell death	Demonstrates potential therapeutic value in the context of highly activated PI3K/Akt pathways	(Rezazadeh et al., 2020; Eum et al., 2011; Kadry et al., 2025)
PI3K/Akt inhibitor	PI3K or Akt inhibitors	Blocking mTOR upstream signaling → Lifting autophagy inhibition	may be converted into autophagy-related cell death or adaptive mechanisms	Demonstrates synergistic effects when combined with PARPi or chemotherapy	(Bian et al., 2018; Philip et al., 2017)
PARPi	Olaparib, etc.	DNA damage and replication stress → Enhanced autophagy	Adaptive autophagy supports drug resistance formation	Inhibiting autophagy enhances PARPi efficacy; affected by HRD and PI3K status	(Santiago-O’Far et al., 2020; Apelian et al., 2025)
ADCs	FRα-targeted ADCs, etc.	The endocytosis-lysosomal pathway activates autophagy	Primarily protective autophagy	Inhibiting autophagy enhances ADC toxicity	(Lee and Liu, 2019; Xue et al., 2024; Chelariu-Raic et al., 2025)
Natural autophagy inducers	curcumin, resveratrol, etc.	Inhibition of AKT/mTOR or activation of AMPK → Enhancement of autophagy	In some models, it converts to autophagy-related cell death	Potential adjunctive treatment strategies; highly context-dependent	(Liu et al., 2019; Yan et al., 2020; Wu et al., 2021; Xie et al., 2021)
Immunotherapy-Related Regulation	Anti-PD-1/PD-L1	Regulating MHC I Presentation and PD-L1 Lysosomal Degradation	Can enhance antigen presentation or support immune evasion	Autophagy regulation may influence immunotherapy response	(Zhan et al., 2022; Cr et al., 2009; Ynebr, 2020; Senba and Mori, 2012; Cui et al., 2023; You et al., 2017; Pan et al., 2016)

## 6 Autophagy and therapeutic strategies

### 6.1 Application of autophagy inhibitors in gynecologic oncology treatment

Research on gynecologic tumor models has confirmed that chemotherapy and targeted therapy are often accompanied by a significant increase in autophagy levels within tumor cells (Zhu et al., 2017). Extensive research indicates that therapy-induced autophagy enables tumor cells to maintain homeostasis by buffering DNA damage, alleviating oxidative stress, and mitigating metabolic stress, thereby allowing them to evade immune-mediated cell death and ultimately promoting the development of a drug-resistant phenotype (Peracchio et al., 2012). Therefore, when autophagy primarily functions as a cell-protective mechanism in therapeutic contexts, targeted inhibition of the autophagy pathway is considered a rational strategy to enhance tumor treatment sensitivity and reverse drug resistance. Currently, the most extensively studied autophagy inhibitors that have advanced to clinical exploration are primarily CQ and HCQ (Xu et al., 2018). In preclinical models of gynecologic tumors, these drugs are rarely utilized alone; instead, they are predominantly combined with platinum-based and taxane chemotherapy agents to attenuate tumor cells’ adaptive response to treatment-induced stress (Pagotto et al., 2017). Previous studies have demonstrated that the combination of CQ or HCQ with cisplatin significantly promotes the accumulation of DNA damage in tumor cells (such as upregulation of phosphorylated histone H2AX expression), activates the mitochondrial-dependent apoptosis pathway, and partially restores tumor cell sensitivity to chemotherapy drugs. This effect is particularly consistent in OC and EC models (Hwang et al., 2020; Nu Ez-Olvera et al., 2019).

While CQ and HCQ represent the most extensively investigated conventional autophagy inhibitors, emerging experimental approaches are being developed to achieve more potent or selective autophagy-targeting effects. Given the multiple limitations of traditional lysosomal inhibitors in clinical applications, research and development of next-generation autophagy inhibitors have garnered significant attention in recent years. Among these, Lys05(Lys01 trihydrochloride), a dimeric derivative of CQ-demonstrates markedly superior lysosomal accumulation capacity and autophagic flux inhibition efficiency compared to CQ/HCQ. When combined with chemotherapeutic agents in multiple solid tumor models, Lys05 exhibits enhanced synergistic antitumor effects (Amaravadi and Winkler, 2012; Jovanovi et al., 2022). Studies have shown that palmitoyl protein thioesterase 1 (PPT1) serves as a key molecular target of CQ derivatives, including Lys05. These agents exert antitumor effects by disrupting the coupling between lysosomal homeostasis and mTOR signaling, thereby regulating upstream pathways involved in autophagy and metabolism (Rebecca et al., 2019). Although research on the aforementioned strategy in gynecologic tumors remains in the preclinical stage, it provides new theoretical foundations and technical pathways for overcoming the efficacy bottlenecks of traditional autophagy inhibitors.

In addition to the aforementioned conventional and emerging autophagy inhibitors, experimental autophagy inhibitors such as Bafilomycin A1 are widely used in basic research to validate the role of autophagy blockade in reversing tumor drug resistance. As specific inhibitors of vacuolar-type ATPase (V-ATPase), these agents disrupt the fusion of autophagosomes with lysosomes and subsequent degradation processes by blocking proton pump activity on lysosomal membranes and inhibiting lysosomal acidification, thereby efficiently suppressing autophagic flux (Wang et al., 2021). Bafilomycin A1 is frequently employed as a research tool in various *in vitro* and animal experimental models. It is combined with chemotherapeutic agents or molecularly targeted drugs to elucidate the role of treatment-induced autophagy in tumor cell survival and the development of drug resistance. These mechanistic studies provide crucial conceptual support for autophagy inhibition as an adjunct therapeutic strategy.

It is noteworthy that the therapeutic efficacy of autophagy inhibitors exhibits significant context dependence, influenced by multiple factors including tumor type, autophagy dependency, molecular background, and treatment stage. Autophagy plays a dual role in cancer therapy: it may support tumor cell survival under therapeutic pressure, thereby promoting drug resistance development; conversely, under specific conditions, it may induce tumor cell death or enhance their immune sensitivity (Bhutia et al., 2013). Therefore, excessive or non-selective inhibition of autophagy may exacerbate treatment-related toxicity or induce the activation of alternative metabolic and survival pathways in tumor cells. Current research perspectives increasingly favor treating autophagy inhibition as a context-dependent component within precision combination therapies rather than a universal anti-tumor intervention applicable to all gynecologic malignancies.

### 6.2 Potential therapeutic effects of autophagy inducers

Although autophagy acts as a protective mechanism in most tumor treatment scenarios, sustained or excessive autophagy activation can also exert tumor-suppressive effects under specific molecular contexts or high-stress conditions. Based on this finding, the potential value of autophagy inducers in gynecologic malignant therapy has gradually gained attention. Abnormal activation of the PI3K/Akt/mTOR signaling axis is highly prevalent in gynecologic malignancies, particularly in ovarian and endometrial carcinomas. Phosphatidylinositol-4,5-bisphosphate 3-kinase catalytic subunit alpha (PIK3CA) mutations, PTEN loss, and Akt overactivation are all closely associated with tumor progression and treatment resistance. Within this molecular context, molecularly targeted drugs—especially mTOR pathway inhibitors—can significantly enhance autophagy activity in tumor cells. Studies indicate that in certain ovarian and endometrial carcinoma models, sustained high autophagy induced by mTOR inhibitors is frequently associated with restricted tumor cell proliferation, disrupted energy metabolism, and organelle dysfunction. This suggests that autophagy can transition from an initial “adaptive protective response” to a “burdensome process” that limits tumor cell survival (Kadry et al., 2025). In this state, autophagy no longer functions solely as a tolerance mechanism for tumor cells but may also participate in autophagy-related cell death processes or amplify other death signals, thereby enhancing the overall therapeutic effect. Notably, rapamycin and its derivatives as mTORC1-specific inhibitors, can both suppress tumor cell proliferation signals and enhance autophagic flux while triggering cell death pathways. This suggests that targeted regulation of mTOR-mediated autophagy processes could serve as a crucial component of antitumor strategies for gynecologic malignancies.

In models of gynecologic malignancies (particularly OC), multiple plant-derived natural compounds have been demonstrated to exert antitumor effects by modulating autophagy-related pathways. Curcumin inhibits the AKT/mTOR/S6K1 pathway and can simultaneously induce autophagy and apoptosis, with the autophagy playing a crucial role in the cell death process (Liu et al., 2019). Resveratrol can upregulate the expression of BECN1 and LC3-IIand enhance autophagy activity by modulating the STAT3/AMPK/mTOR signaling pathway, thereby promoting tumor cell death (Yan et al., 2020). Furthermore, natural compounds such as tanshinone I (Wu et al., 2021) and quercetin (Xie et al., 2021) have been shown to effectively induce autophagy in preclinical tumor models. These findings suggest that plant-derived natural compounds hold promise as potential adjuvant therapeutic agents for targeting autophagy regulation. However, autophagy induction exhibits significant context-dependent effects. In certain tumor models, enhanced autophagy activity may instead provide metabolic buffering for tumor cells, thereby diminishing therapeutic efficacy. This paradox suggests that the clinical application of autophagy inducers requires precise identification of suitable patient populations and combination therapy regimens. Their therapeutic value is more likely to be realized in gynecologic tumors with specific molecular subtypes or drug-resistant backgrounds.

### 6.3 The significance of autophagy markers in personalized therapy

With the deepening development of precision medicine concepts in cancer treatment, the assessment of cellular autophagy status is emerging as a potentially significant tool for predicting treatment efficacy and patient stratification. As a highly regulated intracellular degradation and homeostasis maintenance process, autophagy involves core regulatory molecules including LC3, BECN1, and members of the ATG family. The expression levels of these molecules not only reflect dynamic changes in autophagic flux but are also closely associated with tumor cell response to treatment, the development of drug resistance, and patient clinical prognosis. Clinical studies across multiple cancer types have demonstrated correlations between the expression levels of autophagy-related biomarkers and patient overall survival and PFS (He et al., 2014). For example, the expression levels of BECN1 and LC3 in OC tissues are significantly correlated with tumor type, pathological grade, and prognostic indicators, suggesting that these biomarkers can be used to predict the progression and treatment response of OC (Chen et al., 2020; Jovanovi et al., 2022). Additionally, in other solid tumors, high LC3B expression correlates with improved overall survival in patients, while the prognostic significance of BECN1 varies depending on tumor type and clinical management approaches. Although these findings require further validation through larger-scale, higher-quality clinical studies, existing evidence clearly supports the potential value of autophagy-related proteins as tumor prognostic markers.

From the perspective of personalized therapy, detection of autophagy markers can assist in identifying treatment sensitivity among different patients. For instance, in tumors with high autophagy activity, employing autophagy inhibition strategies can enhance the efficacy of chemotherapy or targeted therapy, thereby improving treatment sensitivity and reducing the risk of drug resistance. Conversely, for tumors with impaired autophagy function accompanied by malignant progression and poor prognosis, enhancing autophagy activity may represent an effective therapeutic approach. Therefore, the expression profile of autophagy markers not only serves as a biological indicator for predicting tumor treatment response but also provides guidance for selecting personalized therapeutic strategies in clinical practice. A currently prominent research focus is the interaction between autophagy and the immune-active TME. The autophagy process not only influences the metabolism and survival of tumor cells themselves but also participates in tumor antigen processing, MHC antigen presentation, and the regulation of immune cell function (You et al., 2017; Pan et al., 2016). For example, autophagy-related organelles may participate in antigen degradation and cross-presentation, thereby regulating the activation status of tumor-infiltrating lymphocytes; autophagy-related molecules (such as BECN1) can influence the polarization direction of TAMs, and studies suggest that modulating autophagy activity may enhance the therapeutic efficacy of immune checkpoint inhibitors. These findings provide a theoretical basis for utilizing autophagy markers as predictive factors for immunotherapy efficacy. Consequently, assessing autophagy status in conjunction with tumor immune phenotype analysis can offer supplementary evidence for precision stratification in gynecologic malignant immunotherapy. In clinical practice, immunohistochemistry is commonly used to detect the expression levels of proteins such as LC3 (particularly LC3-II), BECN1, and p62 in tumor tissues. Combined with autophagic flux dynamic assessment techniques, this approach can more accurately reflect the true state of cellular autophagy.

Recent studies indicate that autophagy-related structures and components can be released into the bloodstream via extracellular vesicles and exosomes. For instance, the enrichment levels of molecules such as LC3-bound autophagosome components, BECN1 fragments, and p62 in exosomes can indirectly reflect autophagy activity within tumor cells (Chen et al., 2022). Quantitative detection of circulating autophagy markers in peripheral blood using liquid-phase chips, immunocapture of exosomes, or mass spectrometry not only enables non-invasive monitoring of tumor autophagy status but also facilitates real-time reference for adjusting personalized treatment strategies when combined with dynamic response assessment during therapy (Hanelova et al., 2023). Furthermore, the combined analysis of circulating autophagy markers with circulating tumor DNA, tumor immunophenotyping, and metabolic markers enables a more comprehensive characterization of tumor autophagy features and their functionally relevant aspects in treatment. This approach lays a more robust foundation for developing precision medicine strategies.

It is important to emphasize that the interpretation of autophagy-related markers at the mechanistic level still has certain limitations. Autophagy is a highly dynamic, stage-continuous cellular process. Detection of molecules such as LC3, BECN1, or p62 at a single time point can only reflect the relative abundance of autophagy-related structures or proteins, and cannot accurately represent the true activity state of autophagic flux. The functional roles of autophagy-related molecules vary across different tumor types and molecular contexts. They may participate in tumor suppression or, under therapeutic pressure, transform into mechanisms promoting tumor cell survival. Furthermore, intratumoral heterogeneity and the temporal evolution of autophagy status during treatment add complexity to inferring overall tumor autophagy dependency based on a single or limited number of autophagy markers. Therefore, at the mechanistic research level, autophagy markers are better suited asindicators of cellular autophagy functional status rather than independent, stable criteria for functional determination. In the future, combined multi-marker analysis, coupled with dynamic autophagy flow assessment methods, and integrated with tumor molecular subtyping and metabolic state analysis, may enable more accurate determination of the true functional positioning of autophagy in individual tumors.

## 7 Discussion

As is well known, autophagy plays a multifaceted role in the etiology, progression, and therapeutic response of gynecologic malignancies, and this review provides a systematic overview of this field. We have placed particular emphasis on the functional significance of autophagy in stress adaptation and the development of treatment resistance. Fundamentally, autophagy is a highly conserved mechanism for maintaining cellular homeostasis, whereby damaged organelles and misfolded proteins are degraded via the lysosomal pathway to generate metabolic substrates that support cell survival under adverse conditions such as hypoxia, nutrient deprivation, or drug exposure. Notably, the functional roles of autophagy differ markedly between normal and tumor cells, as highlighted in this review. Autophagy is tightly regulated by multiple regulatory layers, including the mTOR signaling axis, energy-sensing pathways, transcriptional networks, and the subcellular localization of p53. A comprehensive understanding of the context-dependent nature of autophagy in gynecologic malignancies, including how its functional effects vary with tumor stage, cellular context, molecular background, and therapeutic conditions, is therefore essential for developing context-aware therapeutic strategies that appropriately exploit or restrain autophagy.

At the therapeutic level, modulating autophagy has emerged as a promising intervention strategy for gynecologic malignancies. Existing research confirms that autophagy is frequently activated during platinum-based and taxane chemotherapy, targeted therapy, and immunotherapy, serving as a crucial adaptive mechanism for tumor cells in response to DNA damage, oxidative stress, and metabolic stress (Tam et al., 2021). Based on this mechanism, various autophagy regulation strategies have been proposed, including inhibiting autophagic flux through pharmacological or genetic means, or inducing excessive autophagy under specific conditions to trigger tumor cell death. Preclinical and early clinical studies indicate that autophagy inhibitors such as CQ/HCQ, when combined with chemotherapy or targeted drugs, can improve therapeutic sensitivity and modestly delay the emergence of drug resistance, although clinical data remain limited to small cohorts or preclinical models. This suggests that autophagy represents a potentially modifiable target in regulating tumor treatment response (Mohsen et al., 2022). However, autophagy does not exert a uniform effect across gynecologic malignancies. Its functional outcome may shift between tumor-suppressive and tumor-promoting roles depending on tumor stage, molecular background, the intensity and duration of cellular stress, and therapeutic context.

Although autophagy regulation strategies demonstrate certain clinical application potential, their clinical translation still faces substantial limitations. First, currently available autophagy inhibitors generally lack tumor specificity. While inhibiting autophagy in tumor cells, they may interfere with the basal autophagy processes essential for maintaining homeostasis in normal tissues, thereby posing potential safety concerns (Elshazly et al., 2025). Secondly, the autophagy pathway exhibits high redundancy and dynamic adaptability, making interventions targeting a single pathway prone to limited efficacy due to tumor cell signaling pathway remodeling or secondary drug resistance (Rahdan et al., 2024). Furthermore, the lack of a unified standard for defining “protective autophagy” versus “lethal autophagy” across different studies has, to some extent, increased the complexity of interpreting research findings and facilitating clinical translation (Lim and Murthy, 2020).

Accordingly, autophagy inhibition or induction should not be considered universally applicable strategies, and the appropriate approach may depend on the functional status of autophagy, tumor molecular characteristics, and the specific therapeutic context. Against this backdrop, precise diagnosis and patient stratification are particularly critical for the clinical application of autophagy-targeted therapies. The expression patterns of autophagy-related molecules and signaling pathway components in gynecologic tumors are closely associated with disease progression, treatment response, and patient prognosis, demonstrating potential value as biomarkers. However, given the dynamic nature of autophagy, static expression levels of individual markers, such as LC3, BECN1, and p62, may not accurately reflect the functional status of autophagic flux. Therefore, the combined assessment of multiple autophagy-related markers, together with dynamic autophagy flux analysis, may provide a more reliable evaluation of autophagy activity. Systematically identifying and validating reliable autophagy-related biomarkers may therefore help stratify patients according to autophagy-related characteristics and predict their response to specific autophagy-modulating therapies, providing a basis for biomarker-guided therapeutic strategies and personalized combination treatment regimens. In the future, integrating autophagy functional status, tumor molecular subtyping, and therapeutic strategies holds promise for advancing the precision application of autophagy-targeted interventions in gynecologic malignancies.

Overall, autophagy plays a context-dependent dual role in gynecologic malignancies, with its functional consequences shaped by tumor stage, molecular background, the tumor microenvironment, and therapeutic pressure. The same autophagic machinery may exert tumor-suppressive or tumor-promoting effects depending on the specific cellular and disease context. Therefore, precise assessment of autophagy functional status, together with appropriate therapeutic modulation, may provide a basis for developing personalized treatment strategies for gynecologic malignancies. Further integration of autophagy-related biomarkers, tumor molecular characteristics, microenvironmental features, and therapeutic conditions may facilitate the clinical translation of autophagy-targeted approaches toward precision oncology.

## Glossary

ADCs: Antibody-drug conjugates
ADP: Adenosine diphosphate
Akt: Protein kinase B
AMP: Adenosine monophosphate
AMPK: AMP-activated Protein Kinase
ARID1A: AT-rich interaction domain 1A
ATF4: Activating transcription factor 4
ATG: Autophagy-related
ATP: Adenosine triphosphate
Bcl-2: B-cell lymphoma 2
Bcl-XL: B-cell lymphoma-extra large
BH3: Bcl-2 homology 3
BNIP3: Bcl-2/adenovirus E1B 19 kDa interacting protein 3
BRCA1: Breast cancer type 1 susceptibility protein
CAFs: Cancer-associated fibroblasts
CAMKKβ: Calcium/Calmodulin-Dependent Protein Kinase Kinase Beta
CC: Cervical cancer
cGAS: Cyclic GMP-AMP synthase
CIN: Cervical intraepithelial neoplasia
CMA: Chaperone-mediated autophagy
CQ: Chloroquine
DRAM1: Damage-regulated autophagy modulator 1
E-cadherin: Epithelial Cadherin
EC: Endometrial cancer
eIF2α: Eukaryotic Initiation Factor 2 alpha
EMC-6: ER Membrane Protein Complex Subunit 6
EMT: Epithelial-mesenchymal transition
ER: Endoplasmic reticulum
ERK: Extracellular signal-regulated kinase
FAK: Focal adhesion kinase
FOXO: Forkhead Box O
FOXO1/3: Forkhead box O transcription factors
FRα: Folate receptor alpha
FIGO: International Federation of Gynecology and Obstetrics
GATA4: GATA binding protein 4
HCQ: Hydroxychloroquine
HDAC: Histone deacetylase
HGSOC: High-grade serous ovarian cancer
HIF-1α: Hypoxia-inducible factor 1-alpha
HPV: Human papillomavirus
HR: Homologous recombination
HRD: Homologous recombination deficiency
IL-6: Interleukin-6
IRE1: Inositol-requiring enzyme 1
JNK: c-Jun N-terminal kinase
Keap1: Kelch-like ECH-associated protein 1
LC3: Microtubule-associated Protein 1 Light Chain 3
LC3-II: the lipidated form of microtubule-associated protein 1 light chain 3
MAPK: Mitogen-Activated Protein Kinase
MHC: Major histocompatibility complex
mTOR: Mechanistic Target of Rapamycin
mTORC1: Mechanistic target of rapamycin complex 1
mTORC2: Mechanistic target of rapamycin complex 2
NF-κB: Nuclear Factor Kappa-light-chain-enhancer of Activated B Cells
NIX/BNIP3L: BCL2 Interacting Protein 3 Like
NLRC5: NLR family CARD Domain Containing 5
Nrf2: Nuclear Factor Erythroid 2-related Factor 2
OC: Ovarian Cancer
p62: Sequestosome 1
PARPi: Poly ADP-ribose polymerase inhibitors
PD-1: Programmed cell death protein 1
PD-L1: Programmed cell death-ligand 1
PERK: Protein kinase R-like ER kinase
PFS: Progression-free survival
PI3K: Phosphatidylinositol 3-kinase
PI3P: Phosphatidylinositol 3-phosphate
PTEN: Phosphatase and tensin homolog
RAD51: RAD51 recombinase
Rheb: Ras homolog enriched in brain
ROS: Reactive oxygen species
STAT3: Signal transducer and activator of transcription 3
STIC: Serous tubal intraepithelial carcinoma
STING: Stimulator of interferon genes
SWI/SNF: SWItch/sucrose non-fermentable
TAMs: Tumor-associated macrophages
TCA: Tricarboxylic acid cycle
TFE3: Transcription factor binding to IGHM enhancer 3
TFEB: Transcription factor EB
TME: Tumor microenvironment
TP53: Tumor protein 53
ULK1: Unc-51-like autophagy-activating kinase 1
UPR: Unfolded protein response
V-ATPase: Vacuolar-type ATPase
VEGF: Vascular endothelial growth factor
VPS34: Vacuolar protein sorting 34
BECN1: Beclin-1
**CD8** ^**+**^ **T cells**: CD8^−^positive T lymphocytes
DNA: Deoxyribonucleic acid
MHC-I: Major histocompatibility complex class I
Lys05: Lys01 trihydrochloride
p65: Nuclear factor NF-kappa-B p65 subunit
SGK1: Serum/glucocorticoid-regulated kinase 1
PKC: Protein kinase C
mSIN1: Mammalian stress-activated protein kinase-interacting protein 1
Rictor: Rapamycin-insensitive companion of mTOR
Protor: Protein observed with Rictor
